# Remotely delivered cognitive behavioural and personalised exercise interventions for fatigue severity and impact in inflammatory rheumatic diseases (LIFT): a multicentre, randomised, controlled, open-label, parallel-group trial

**DOI:** 10.1016/S2665-9913(22)00156-4

**Published:** 2022-06-27

**Authors:** Eva-Maria Bachmair, Kathryn Martin, Lorna Aucott, Neeraj Dhaun, Emma Dures, Richard Emsley, Stuart R Gray, Elizabeth Kidd, Vinod Kumar, Karina Lovell, Graeme MacLennan, Paul McNamee, John Norrie, Lorna Paul, Jon Packham, Stuart H Ralston, Stefan Siebert, Alison Wearden, Gary Macfarlane, Neil Basu, LIFT study group

**Affiliations:** a Aberdeen Centre for Arthritis and Musculoskeletal Health, University of Aberdeen, Aberdeen, UK; b Centre of Healthcare and Randomised Trials (CHaRT), Health Service Research Unit, University of Aberdeen, Aberdeen, UK; c Health Economics Research Unit, University of Aberdeen, Aberdeen, UK; d Institute of Cardiovascular and Medical Sciences, BHF Glasgow Cardiovascular Research Centre, University of Glasgow, Glasgow, UK; e Institute of Infection, Immunity and Inflammation, University of Glasgow, Glasgow, UK; f British Heart Foundation Centre of Research Excellence, Centre for Cardiovascular Science, The Queen's Medical Research Institute, Edinburgh, UK; g Edinburgh Clinical Trials Unit, Western General Hospital, University of Edinburgh, Edinburgh, UK; h Rheumatology and Bone Disease, Western General Hospital, University of Edinburgh, Edinburgh, UK; i Faculty of Health and Applied Sciences, University of the West of England, Bristol, UK; j Department of Biostatistics and Health Informatics, Institute of Psychiatry, Psychology and Neuroscience, King's College London, London, UK; k Department of Rheumatology, Freeman's Hospital, The Newcastle upon Tyne Hospitals NHS Foundation Trust, Newcastle upon Tyne, UK; l Department of Rheumatology, Ninewells Hospital, NHS Tayside, Dundee, UK; m School of Health Sciences, University of Manchester, Manchester, UK; n School of Health and Life Science, Glasgow Caledonian University, Glasgow, UK; o Physiotherapy and Paramedicine, Haywood Rheumatology Centre, Stoke-on-Trent, UK

## Abstract

**Background:**

Chronic fatigue is a poorly managed problem in people with inflammatory rheumatic diseases. Cognitive behavioural approaches (CBA) and personalised exercise programmes (PEP) can be effective, but they are not often implemented because their effectivenesses across the different inflammatory rheumatic diseases are unknown and regular face-to-face sessions are often undesirable, especially during a pandemic. We hypothesised that remotely delivered CBA and PEP would effectively alleviate fatigue severity and life impact across inflammatory rheumatic diseases.

**Methods:**

LIFT is a multicentre, randomised, controlled, open-label, parallel-group trial to assess usual care alongside telephone-delivered CBA or PEP against usual care alone in UK hospitals. Patients with any stable inflammatory rheumatic disease were eligible if they reported clinically significant, persistent fatigue. Treatment allocation was assigned by a web-based randomisation system. CBA and PEP sessions were delivered over 6 months by trained health professionals in rheumatology. Coprimary outcomes were fatigue severity (Chalder Fatigue Scale) and impact (Fatigue Severity Scale) at 56 weeks. The primary analysis was by full analysis set. This study was registered at ClinicalTrials.gov (NCT03248518).

**Findings:**

From Sept 4, 2017, to Sept 30, 2019, we randomly assigned and treated 367 participants to PEP (n=124; one participant withdrew after being randomly assinged), CBA (n=121), or usual care alone (n=122), of whom 274 (75%) were women and 92 (25%) were men with an overall mean age of 57·5 (SD 12·7) years. Analyses for Chalder Fatigue Scale included 101 participants in the PEP group, 107 in the CBA group, and 107 in the usual care group and for Fatigue Severity Scale included 101 in PEP, 106 in CBA, and 107 in usual care groups. PEP and CBA significantly improved fatigue severity (Chalder Fatigue Scale; PEP: adjusted mean difference −3·03 [97·5% CI −5·05 to −1·02], p=0·0007; CBA: −2·36 [–4·28 to −0·44], p=0·0058) and fatigue impact (Fatigue Severity Scale; PEP: −0·64 [–0·95 to −0·33], p<0·0001; CBA: −0·58 [–0·87 to −0·28], p<0·0001); compared with usual care alone at 56 weeks. No trial-related serious adverse events were reported.

**Interpretation:**

Telephone-delivered CBA and PEP produced and maintained statistically and clinically significant reductions in the severity and impact of fatigue in a variety of inflammatory rheumatic diseases. These interventions should be considered as a key component of inflammatory rheumatic disease management in routine clinical practice.

**Funding:**

Versus Arthritis

## Introduction

Inflammatory rheumatic diseases comprise the majority of a rheumatologist's workload and include chronic immune-mediated disorders such as rheumatoid arthritis, axial spondyloarthritis, and systemic lupus erythematosus. Inflammatory rheumatic diseases are common conditions, with an overall lifetime risk of approximately 8·4% for women and 5·1% for men,^1^ and are major contributors to the global disability burden.^2^

The symptom of fatigue is a shared burden across inflammatory rheumatic diseases. Despite substantial advances in therapeutics, as many as 80% of patients report fatigue and over 70% consider fatigue to be as important as pain.^3^, ^4^ Fatigue is a major determinant of impaired quality of life and a principal predictor of work disability.^5^

Although there are no evidence-based pharmacological interventions for inflammatory rheumatic disease-related fatigue, a Cochrane review of non-pharmacological interventions^6^ reported substantial benefits of psychosocial and physical activity interventions in reducing fatigue among patients with rheumatoid arthritis. However, health-care services encounter multiple barriers to their implementation. First, the testing of previous interventions was limited to single diseases in isolation, and so they are not appropriate if the clinically diverse inflammatory rheumatic diseases served by a rheumatology service are to receive equitable care. There has been a shift towards conceptualising fatigue as a generic symptom with shared person-specific factors across conditions rather than predominating disease-specific factors.^7^ However, non-pharmacological interventions for fatigue have not been tested across inflammatory rheumatic disease diagnoses or any other chronic diseases. Second, specialist expertise, such as clinical psychology, is not easily accessible and does not often exist within speciality multidisciplinary teams.^8^ Third, some patients find it challenging to attend regular face-to-face treatment sessions due to a combination of their health, transport issues, and family or work commitments.^9^ Moreover, the safety benefits of remote care delivery have been highlighted during the COVID-19 pandemic. As a result of the COVID-19 pandemic, health-care systems are increasingly encouraging long-term adoption of remotely delivered services. However, the effectiveness of such approaches has been insufficiently tested in multiple specialities, including rheumatology.

**Research in context**

**Evidence before this study**

Chronic fatigue is common and considered a principal burden by patients with inflammatory rheumatic disease, even those who have attained pharmacological disease remission. International clinical guidelines do not currently specify fatigue management recommendations for this large clinical population. We searched PubMed, Scopus, and Cochrane Database of Systematic Reviews for clinical trials with the search terms “fatigue”, “rheumatoid arthritis”, “arthritis”, “spondyloarthritis”, “vasculitis”, “rheumatology”, “lupus”, “ankylosing spondylitis”, “Sjögren”, “scleroderma”, “connective tissue disease”, “psoriatic arthritis” for literature published between March 1, 2015, and June 1, 2021, with no language restrictions. A 2013 Cochrane systematic review reported significant fatigue reductions from physical activity and psychosocial interventions in rheumatoid arthritis but did not identify any high-quality studies (assessed by Cochrane's risk of bias quality component tools). Since then, a single, high-quality study with identical methods to the Cochrane systematic review has been reported, providing evidence that cognitive behavioural approaches reduce fatigue impact in rheumatoid arthritis when delivered face-to-face by trained members of the rheumatology multidisciplinary team. However, no trials have evaluated the generic fatigue alleviating effect of non-pharmacological interventions in a mixed population of patients with inflammatory rheumatic diseases (representative of a typical rheumatology service client cohort) nor have they examined efficient methods of intervention delivery (eg, remote delivery), which might facilitate implementation.

**Added value of this study**

This fatigue alleviation trial is the first to test non-pharmacological interventions in a range of inflammatory rheumatic diseases and the first to evaluate their remote delivery of care by trained members of the rheumatology multidisciplinary team. Both telephone-delivered physical exercise interventions and cognitive behavioural interventions provided clinically and statistically significant improvements in fatigue severity and impact across a generalisable population of patients with inflammatory rheumatic disease. These effects were maintained 6 months following intervention cessation.

**Implications of all the available evidence**

Taken together, physical activity and psychosocial interventions that have been specifically developed are effective in alleviating fatigue in patients with inflammatory rheumatic diseases and should be recommended in routine clinical practice. Their generic delivery across inflammatory rheumatic diseases by trained members of the speciality team should reduce barriers to health service implementation. Moreover, their remote delivery offers opportunities for time efficiencies for both care providers and patients as well as safety during pandemic conditions.

In this Lessening the Impact of Fatigue in Inflammatory Rheumatic Disease Trial (LIFT) study, we aimed to identify whether psychosocial and physical activity interventions, delivered by telephone by the rheumatology multidisciplinary teams, were clinically effective and safe in improving fatigue for patients who were otherwise stable across the inflammatory rheumatic disease spectrum. We hypothesised that up to eight sessions (over 22 weeks) of either a standardised cognitive behavioural approach plus usual care (CBA) or a personalised exercise programme plus usual care (PEP), would be more effective than usual care alone to reduce the impact and severity of fatigue after a 56 week follow-up period.

## Methods

### Study design and participants

LIFT was a multicentre, randomised, controlled, open-label, parallel-group trial. The trial protocol has been previously published^10^ and subsequent amendments are reported in the appendix (pp 18–21). We recruited patients attending six secondary care rheumatology services in England and Scotland. We considered participants if they were 18 years or older at the time of consent, had been diagnosed with an inflammatory rheumatic disease by a consultant rheumatologist, and reported fatigue to be a problem that was both persistent (>3 months) and clinically significant (≥6 on numerical rating 0–10 scale measuring average level of fatigue during the past 7 day). Participants were excluded if they had unstable inflammatory disease (as evidenced by changed immunomodulatory therapy in the previous 3 months), a potential explanation for fatigue that was medically reversible (eg, severe anaemia), or a medical condition that would make the proposed interventions unsuitable (eg, clinically significant heart disease). The complete list of inclusion and exclusion criteria is in the appendix (p 2).

Written informed consent was obtained from all patients before any study-related procedures were done at the baseline visit. The trial was approved by the Research Ethics Committee Wales 7 (17/WA/065) and the research and development departments of each participating NHS health board or trust and conducted in accordance with the Declaration of Helsinki, the International Conference on Harmonization Good Clinical Practice guidelines, and UK regulations.

### Randomisation and masking

Participants were allocated to receive either PEP, CBA, or usual care (1:1:1 ratio) using a computer-generated sequence that was accessed remotely via a web-based randomisation system. Randomisation was minimised by diagnosis (rheumatoid arthritis, systemic lupus erythematosus, axial spondyloarthritis, or other inflammatory rheumatic diseases) and the presence or absence of depressive symptoms (Hospital Anxiety and Depression Scale subscale score >10).^11^ The minimisation algorithm included a 20% random twist—ie, 20% of all the allocated randomisations were randomly reallocated 1:1 to the remaining two treatment options. Randomisation was done by research nurses in the recruiting centres employing the trial's custom-built database, which included the randomisation tool, electronic case report form, and safety reporting. Full masking was not possible due to the nature of the interventions, which required active engagement of participants and therapists. All investigators, including statisticians, were masked to treatment allocation.

### Procedures

All participants were aware that the trial interventions were designed specifically to reduce fatigue. As a minimum, all participants received usual care in the form of a Versus Arthritis (formerly Arthritis Research UK) education booklet for fatigue. This booklet addresses the principal domains of fatigue, which can be amenable to self-management and represents usual care in almost all rheumatology services in the UK.

The CBA and PEP active treatments were therapist based, with accompanying manuals. They were adapted, with patient involvement, from previous fatigue-specific cognitive behavioural and exercise interventions^12^, ^13^ to ensure that they were suitable for remote delivery via telephone, and were applicable to the broad spectrum of inflammatory rheumatic diseases. A detailed description of each intervention is available in the appendix (pp 3–8). Briefly, CBA was a psychological intervention that targeted unhelpful beliefs and behaviours and aimed to replace them with more adaptive ones. PEP was an exercise programme that was individually tailored and combined with a graded exposure behavioural therapy that was aimed to normalise misperceptions of effort and enhance exercise tolerance.

Both CBA and PEP interventions were delivered by telephone by health professionals in rheumatology employed within local NHS rheumatology departments. These health professionals received intensive training and supervision from experienced exercise therapists or a cognitive behavioural therapist and clinical psychologist with expertise in fatigue interventions. They were additionally supported throughout the study, with therapist manuals and ongoing individual supervision. Based on previous trials,^13^ participants were offered a maximum of seven one-to-one sessions, each up to 45 mins in duration, over 14 weeks with a booster session done at 22 weeks after the start of the intervention. The final number of sessions was individually determined between patient and therapist.

Participants were separately asked to attend local clinical research facilities for assessment of outcomes on average at 10 weeks, 28 weeks, and 56 weeks after randomisation. If participants were unable to attend in person, the follow-up was done by telephone by research personnel at the site or centrally by trial office staff. During the COVID-19 pandemic, follow-up was limited to telephone contact and outcomes at 56 weeks were prioritised.

### Outcomes

Our tested interventions were designed to reduce both the severity and impact of fatigue, which are distinct aspects of similar patient importance. Therefore, we collected two primary outcomes: Chalder Fatigue Scale (0 [low] to 33 [high], Likert scale),^14^ a measure of fatigue severity, and the Fatigue Severity Scale (1 [low] to 9 [high] scale),^15^ an assessment of fatigue impact.

Secondary outcomes were multidimensional aspects of fatigue (Bristol Rheumatoid Arthritis Fatigue Multidimensional Questionnaire),^16^ health-related quality of life (Short Form 12),^17^ pain intensity (numerical rating 0–10 scale),^18^ sleep disturbance (Jenkins Sleep Scale),^19^ anxiety and depression (Hospital Anxiety and Depression Scale),^11^ impact on work and activities (Work Productivity and Activity Impairment),^20^, ^21^ and change in global health status.

Adverse events were recorded by local and central study teams after a study-specific standard operating procedure for adverse events in non-clinical trials of investigational medicinal products studies. Events were identified by members of the local research team by asking the participant during assessment visits or during telephone contact with the therapist delivering the intervention whether a potential serious adverse event (SAE) has occurred since the previous contact. In addition, participants self-reported events via direct contact with the local research team, therapist, and by completion of study questionnaires (but also during telephone calls with the trial office staff). Adverse events were then assessed for seriousness and relatedness by a designated experienced investigator with rheumatology expertise (NB) and investigators responsible for the training of therapists, as required.

### Statistical analysis

Our planned primary analysis strategy was to separately compare CBA plus usual care with usual care alone, and PEP plus usual care with usual care alone. To preserve the overall 5% error with two comparisons and two primary outcomes tested sequentially, we designated the Chalder Fatigue Scale at 56 weeks as the dominant primary outcome and only if positive would the Fatigue Severity Scale then be formally analysed.

The clinically minimally important effect was 0·5,^22^ equating to 2 points in the Chalder Fatigue Scale (assuming SD of 4 points), based on the trials that evaluated similar non-pharmaceutical interventions. The prespecified alpha for these two comparisons was set at 2·5% to maintain an overall alpha of 5**%**. For 90% power, we required 100 evaluable participants in each of the three groups. From our own previous trials, we expected a dropout rate of 20% and inflated the target sample size to 125 participants in each treatment group or 375 participants in all.

We used a simple *t* test approach but planned and used repeated measures ANCOVA regression models to increase precision by adjusting for the baseline analogue of the primary outcome measures, using serial measures at three follow-up time points, and including baseline predictors (used in the minimisation procedure). A factor that we anticipated to decrease power was any potential clustering due to any therapist effects (the health professionals in rheumatology delivering either the PEP or the CBA intervention). We expected that any such clustering would be small, especially given the primary timepoint of interest was at 56 weeks. Given the difficulties in specifying relevant intraclass correlation coefficients (ICC), the methodological difficulties in the sample size to adjust for this in two of the groups (with possibly different ICC), and not in the usual care group, and the subsequent uneven allocation ratios to optimise power that arise from such calculations, we did not explicitly adjust for therapist ICCs in the sample size calculation. We did expect any gains in power from using baseline and repeated within-person measures to offset any small loss in power arising from potential therapist effects.

Continuous variables were summarised using mean (SD) and discrete variables and were reported as absolute numbers and proportions. The primary outcomes were analysed using a heteroscedastic partially-nested repeated measures mixed-effects linear model. This model included the baseline version of the score and binary fixed effects variables for scoring more than 10 on the Hospital Anxiety and Depression Scale depression subscale. Treatment effects were estimated from the treatment-by-time interaction and the main timepoint of interest was 56 weeks. A random effect for therapist was included in the CBA group only to incorporate clustering due to therapist effect, there was no evidence of therapist effect in the PEP group, a random effect for the centre was included for the PEP and control group. Degrees of freedom were adjusted for the small number of clusters using the Kenward-Rogers method. The primary approach used all follow-up data and analysed-as-randomised approach under a missing-at-random assumption, a full analysis set analysis. Imputation and pattern mixture models were used to test the robustness of intervention effect estimates under different assumptions, these are described and reported in detail in the appendix (p 9 and pp 11–12). Additional analyses done for the primary outcomes were: complier average causal effect to estimate the intervention effect in complies (prespecified); a post-hoc subgroup analysis by diagnosis (rheumatoid arthritis *vs* non-rheumatoid arthritis) at baseline; a post-hoc subgroup analysis by gender; and the impact on patients whose outcomes might have been influenced by COVID-19-related lockdowns (prespecified). We also did a post-hoc comparison of PEP versus CBA. These analyses are described in more detail in the appendix (pp 16–18). The primary outcomes are reported using 97·5% CIs to reflect the two comparisons with control. Secondary outcomes were analysed using similar models but reported with 95% CIs and there were no multiplicity adjustments made to secondary outcomes. All analyses used Stata (version 16.0). A cost-effectiveness analysis will be reported separately. This study was prospectively registered with ClinicalTrials.gov, NCT03248518. Analyses in the statistical analysis plan not reported in this Article will be reported in future publications.

### Role of the funding source

The funder had no role in data collection, data analysis, data interpretation, or writing of the manuscript.

## Results

**Figure 1:**
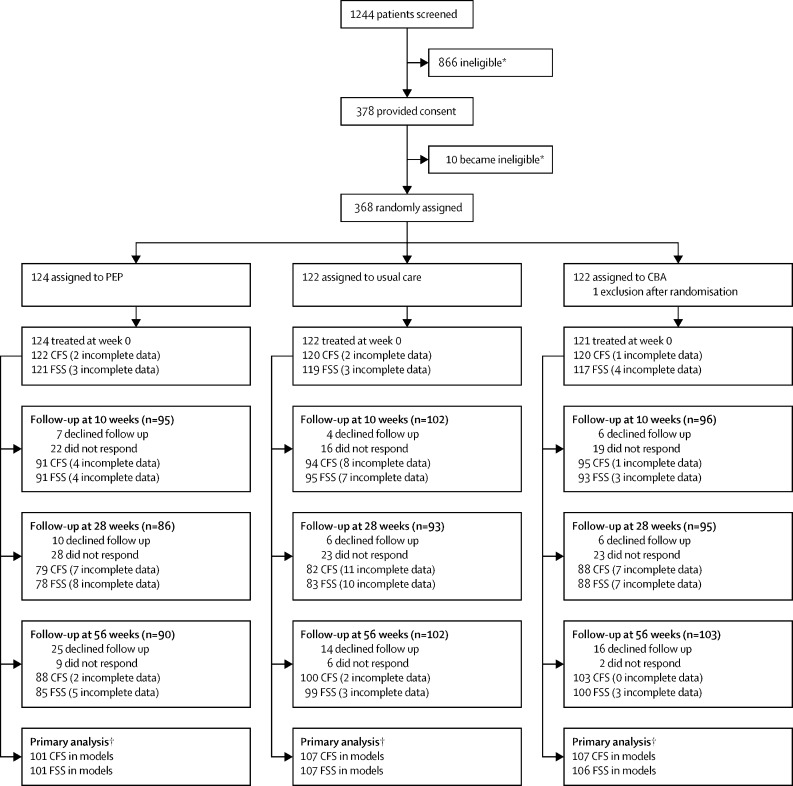
Trial profile Non-responders refers to participants given the opportunity to report but for whom there was no data. CFS=Chalder Fatigue Scale. FSS=Fatigue Severity Score. PEP=personalised exercise programme. CBA=cognitive behavioural approaches. *Reasons for ineligibility are listed in the appendix (p 10). †Includes any participant into the analysis for the primary outcome mixed model who had a baseline record and data for at least one of the follow-up points.

We identified 1244 potentially eligible participants, of whom 378 (30%) met the criteria for additional assessment and consented between Sept 4, 2017, and Sept 30, 2019. The final participant visit was on Oct 31, 2020. Eligibility was confirmed in 368 (97%) patients, allocated to either PEP (n=124), CBA (n=122), or usual care (n=122; figure 1). One participant withdrew after being randomly assinged in the PEP group and one participant was excluded from the CBA group after being randomly assigned because a recent change in immunosuppressive medication had been discovered.

Of those treated, 202 (55%) were diagnosed with rheumatoid arthritis, 78 (21%) with connective tissue disease, 72 (20%) with axial spondyloarthritis, 14 (4%) with other inflammatory rheumatic diseases, and 1 requested their data to be withdrawn. Overall, 274 (75%) participants were female and 92 (25%) were male. Participants had a mean (SD) age of 57·0 (12·7) years, disease duration of 11·4 (10·2) years, and low levels of systemic inflammation (erythrocyte sedimentation rate: 16·2 [15·9] mm/h). The groups were balanced across baseline characteristics (table 1).

**Table 1:** Baseline characteristics of study participants

		PEP group (n=124)*	**CBA group (n=121)**	**Usual care group (n=122)**
Mean age, years		56·4 (12·3)	59·3 (13·0)	56·8 (12·7)
Gender				
	Female	97 (78%)	84 (69%)	93 (76%)
	Male	26 (21%)	37 (31%)	29 (24%)
	Missing data	1 (1%)	0	0
Employment Group				
	Working fulltime (≥30 h per week)	35 (28%)	36 (30%)	38 (31%)
	Working part-time (<30 h per week)	16 (13%)	16 (13%)	23 (19%)
	Unemployed and looking for work	2 (2%)	1 (1%)	1 (1%)
	Unable to work because of illness or disability	20 (16%)	14 (12%)	16 (13%)
	Homemaker and not looking for paid employment	4 (3%)	2 (2%)	3 (2%)
	Student	2 (2%)	2 (2%)	1 (1%)
	Retired	42 (34%)	46 (38%)	36 (30%)
	Other	2 (2%)	3 (2%)	2 (2%)
	Missing data	1 (1%)	1 (1%)	2 (2%)
Ethnicity				
	Scottish	87 (70%)	87 (72%)	97 (80%)
	Other British	27 (22%)	25 (21%)	21 (17%)
	Irish	0	1 (1%)	0
	Other White	7 (6%)	5 (4%)	1 (1%)
	Other Ethnic (Arabic)	1 (1%)	0	0
	Missing data	2 (2%)	3 (2%)	3 (2%)
Centre				
	One	50 (40%)	49 (40%)	50 (41%)
	Two	23 (19%)	24 (20%)	24 (20%)
	Three	14 (11%)	13 (11%)	13 (11%)
	Four	22 (18%)	21 (17%)	21 (17%)
	Five	9 (7%)	10 (8%)	10 (8%)
	Six	6 (5%)	4 (3%)	4 (3%)
Diagnosis				
	Rheumatoid arthritis	67 (54%)	67 (55%)	68 (56%)
	Axial spondyloarthritis	25 (20%)	24 (20%)	23 (19%)
	Connective tissue disease	26 (21%)	27 (22%)	25 (20%)
	Other†	5 (4%)	3 (2%)	6 (5%)
	Missing data	1 (1%)	0	0
Median disease duration, years		8·5 (3·6–14·9)	8·7 (2·7–15·9)	9·3 (3·2–17·5)
	Missing data	33 (27%)	24 (20%)	31 (25%)
Median Charlson index score (other comorbidities; IQR)		1 (1–2)	1 (1–2)	1 (1–2)
	Missing data	1 (1%)	0	0
Median erythrocyte sedimentation rate, mm/h		13 (17–22)	12 (6–23)	10 (5–17)
	Missing data	3 (2%)	4 (3%)	1 (1%)
Median disease activity self-report, NRS 0–10		6 (4–7)	6 (4–7·5)	5 (4–7)
	Missing data	1 (1%)	1 (1%)	1 (1%)
Median physical activity self-report, days per week		3 (1–5)	3 (1–5)	2 (0–4)
	Missing data	1 (1%)	4 (3%)	2 (2%)
Mean fatigue average score self-report, NRS 0–10		7·4 (1·1)	7·3 (1·0)	7·3 (1·1)
	Missing data	1 (1%)	0	0

Data are n (%), mean (SD), or median (IQR). CBA=cognitive behavioural approaches. PEP=personalised exercise programmes. NRS=numerical rating scale.

* One participant withdrew after treatment in the PEP group and requested removal of their data.

† Systemic vasculitis; synovitis, acne, pustulosis, hyperostosis, and osteitis syndrome; and juvenile inflammatory arthritis, and undifferentiated inflammatory arthritis.

Participants assigned to PEP received a median of five sessions (IQR 1–8) within the 30 week treatment window. In total, 19 (15%) of 124 participants elected to stop PEP within the 30 week treatment window and 20 (16%) did not attend any sessions. There were 14 PEP therapists who saw a median of 12 (range 1–23) patients each. Participants assigned to CBA received a median of eight sessions (IQR 2–8) within the 30 week treatment window. In the CBA group, 11 (9%) participants elected to stop therapy within the 30 week treatment window and 18 (15%) did not attend any CBA sessions. There were 13 CBA therapists who saw a median of 15 patients each (range 1–21). At 56 weeks, 25 participants allocated to PEP, 16 participants allocated to CBA, and 14 participants allocated to usual care declined follow-up, and nine participants allocated to PEP, two allocated to CBA, and six allocated to usual care were lost to follow-up (figure 1). The analysis of Chalder Fatigue Scale included 101 participants in the PEP group, 107 in the CBA group, and 107 in usual care group. For Fatigue Severity Scale analysis, there were 101 participants in the PEP group, 106 in the CBA group, and 107 in the usual care group. The baseline demographic characteristics of the 72 participants whose primary outcomes were not captured were similar to the overall trial population (appendix p 10).

**Figure 2:**
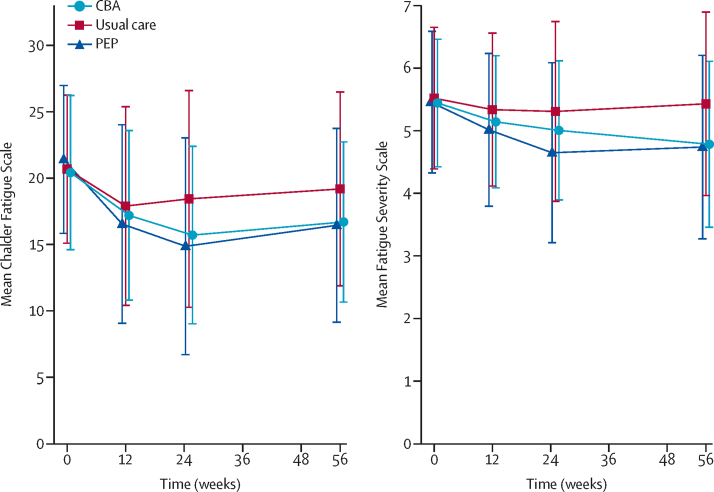
Primary outcomes across follow-up points Error bars show SD. CFS=Chalder Fatigue Scale. FSS=Fatigue Severity Scale. PEP=personalised exercise programmes. CBA=cognitive behavioural approaches.

Chalder Fatigue Scale and Fatigue Severity Scale scores improved over time in both intervention groups (figure 2 and table 2). At 56 weeks, both PEP and CBA reduced fatigue severity measured by the Chalder Fatigue Scale (PEP: adjusted mean difference −3·03 [97·5% CI −5·05 to −1·02], p=0·0007; CBA: −2·36 [–4·28 to −0·44], p=0·0058), and fatigue impact measured by the Fatigue Severity Scale (PEP: −0·64 [–0·95 to −0·33], p<0·0001; CBA: −0·58 [–0·87 to −0·28], p<0·0001) compared with usual care. These differences were equivalent to a fatigue severity effect size of −0·52 (97·5% CI −0·88 to −0·16) for PEP and −0·42 (−0·77 to −0·07) for CBA, and a fatigue impact effect size of −0·63 (−0·93 to −0·32) for PEP and −0·57 (−0·86 to −0·28) for CBA, using the standardised mean difference scale. Multiple imputation sensitivity analyses gave similar results and remained significant even in the most conservative scenario in which missing data from the active treatment groups were assumed to remain unchanged, by contrast to the usual care alone comparator in which the observed intention-to-treat improvements were assumed (at 56 weeks Chalder Fatigue Scale: PEP −1·53 [–3·01 to −0·05] and CBA −1·76 [–3·25 to −0·27]; at 56 weeks Fatigue Severity Scale: PEP −0·43 [–0·69 to −0·17] and CBA −0·43 [–0·69 to −0·17]; appendix pp 11–12). The adjustment for participants receiving at least three sessions of active treatment enhanced the effect size of PEP on fatigue severity (Chalder Fatigue Scale mean difference −4·44 [97·5% CI −5·66 to −3·21], p<0·0001), but had no impact on the treatment effect of PEP on fatigue impact or CBA effect size on either primary outcome (appendix p 16).

**Table 2:** Primary and secondary outcomes of study participants (full analysis set)

	**PEP**	**CBA**	**Usual care**	PEP versus usual care, adjusted mean difference (CI*)	**p value**	CBA versus usual care, adjusted mean difference (CI*)	**p value**
**Chalder Fatigue Scale**							
Baseline	21·4 (5·6); 122	20·4 (5·8); 120	20·7 (5·2); 120	..	..	..	..
10 weeks	16·5 (7·5); 91	17·2 (6·4); 95	17·9 (6·2); 94	−1·70 (−3·72 to 0·32)	0·059	−0·68 (−2·66 to 1·29)	0·44
28 weeks	14·9 (8·2); 79	15·7 (6·7); 88	18·4 (5·7); 82	−3·89 (−6·03 to −1·75)	<0·0001	−2·73 (−4·79 to −0·68)	0·029
56 weeks	16·5 (7·3); 88	16·7 (6·0); 103	19·2 (5·9); 100	−3·03 (−5·05 to −1·02)	0·0007	−2·36 (−4·28 to −0·44)	0·0058
**Fatigue Severity Scale**							
Baseline	5·5 (1·1); 121	5·4 (1·0); 117	5·5 (0·9); 119	..	..	..	..
10 weeks	5·0 (1·2); 91	5·1 (1·1); 93	5·3 (1·1); 95	−0·26 (−0·57 to 0·04)	0·055	−0·11 (−0·41 to 0·20)	0·44
28 weeks	4·7 (1·4); 78	5·0 (1·1); 88	5·3 (1·1); 83	−0·54 (−0·87 to −0·22)	0·0002	−0·24 (−0·55 to 0·08)	0·090
56 weeks	4·7 (1·5); 85	4·8 (1·3); 100	5·4 (1·1); 99	−0·64 (−0·95 to −0·33)	<0·0001	−0·58 (−0·87 to −0·28)	<0·0001
**HADS anxiety subscale**							
Baseline	8·9 (4·4); 123	8·7 (4·5); 121	8·3 (4·2); 122	..	..	..	..
10 weeks	8·6 (4·4); 89	8·6 (4·7); 92	7·9 (4·3); 95	0·13 (−0·74 to 0·99)	0·77	0·31 (−0·55 to 1·18)	0·48
28 weeks	7·5 (5·0); 77	7·9 (4·6); 88	7·8 (4·2); 83	−0·56 (−1·46 to 0·35)	0·23	−0·16 (−1·05 to 0·73)	0·73
56 weeks	7·6 (4·9); 73	7·8 (4·4); 86	7·8 (4·6); 85	−0·74 (−1·65 to 0·17)	0·11	−0·34 (−1·23 to 0·55)	0·45
**HADS depression subscale**							
Baseline	7·3 (3·8); 123	7·1 (3·8); 121	6·8 (3·7); 122	..	..	..	..
10 weeks	7·2 (4·2); 91	6·9 (4·2); 93	6·5 (3·7); 95	0·26 (−0·50 to 1·03)	0·50	0·09 (−0·66 to 0·84)	0·82
28 weeks	5·8 (4·1); 78	6·4 (3·6); 88	6·3 (3·5); 83	−0·70 (−1·51 to 0·10)	0·086	−0·28 (−1·05 to 0·50)	0·49
56 weeks	5·9 (3·9); 75	6·5 (3·8); 88	6·8 (4·0); 85	−1·03 (−1·84 to −0·23)	0·012	−0·47 (−1·24 to 0·30)	0·23
**Short Form-12 physical component summary**							
Baseline	34·7 (9·8); 117	34·1 (10·3); 116	33·4 (10·1); 117	..	..	..	..
10 weeks	36·8 (9·7); 88	35·0 (10·0); 92	33·9 (10·9); 95	1·09 (−0·89 to 3·06)	0·28	0·82 (−1·18 to 2·82)	0·42
28 weeks	36·3 (10·6); 73	34·6 (9·8); 85	34·1 (10·5); 81	0·68 (−1·43 to 2·80)	0·53	0·72 (−1·37 to 2·81)	0·50
56 weeks	36·5 (10·6); 73	34·8 (10·6); 87	33·2 (10·8); 79	1·33 (−0·80 to 3·45)	0·22	0·06 (−2·03 to 2·15)	0·95
**Short Form-12 mental component summary**							
Baseline	40·8 (11·3); 117	41·6 (11·2); 116	42·9 (11·2); 117	..	..	..	..
10 weeks	42·3 (11·1); 88	44·3 (11·0); 92	44·9 (9·5); 95	−1·16 (−3·48 to 1·15)	0·33	0·15 (−2·17 to 2·47)	0·90
28 weeks	45·3 (12·3); 73	45·0 (11·2); 85	44·7 (10·2); 81	1·82 (−0·66 to 4·29)	0·15	0·39 (−2·03 to 2·82)	0·75
56 weeks	44·8 (10·5); 73	45·3 (10·7); 87	43·2 (11·2); 79	2·79 (0·31 to 5·28)	0·027	2·47 (0·04 to 4·89)	0·046
**Bristol Rheumatoid Arthritis Fatigue Multidimensional Questionnaire total score**							
Baseline	41·3 (14·2); 122	38·9 (13·2); 119	40·0 (12·2); 120	..	..	..	..
10 weeks	34·4 (16·6); 91	34·8 (13·8); 94	35·4 (14·2); 95	−2·14 (−5·64 to 1·36)	0·23	0·39 (−3·16 to 3·94)	0·83
28 weeks	31·1 (17·4); 76	33·4 (14·2); 89	34·5 (13·8); 81	−5·07 (−8·76 to −1·38)	0·0070	−0·76 (−4·42 to 2·89)	0·68
56 weeks	31·2 (18·4); 78	30·8 (14·9); 92	36·9 (14·2); 87	−6·99 (−10·63 to −3·34)	<0·0002	−4·93 (−8·53 to −1·33)	0·0073
**Pain (NRS)**							
Baseline	5·9 (2·5); 121	5·7 (2·3); 119	5·8 (2·3); 120	..	..	..	..
10 weeks	5·1 (2·7); 91	5·4 (2·4); 93	5·3 (2·6); 94	−0·27 (−0·83 to 0·29)	0·35	0·02 (−0·65 to 0·69)	0·95
28 weeks	4·8 (2·9); 77	5·3 (2·2); 87	5·2 (2·3); 83	−0·57 (−1·16 to 0·03)	0·063	−0·07 (−0·76 to 0·61)	0·83
56 weeks	5·2 (2·7); 79	5·3 (2·4); 93	5·3 (2·79); 92	−0·26 (−0·84 to 0·32)	0·39	0·15 (−0·52 to 0·82)	0·66
**Jenkins Sleep Scale**							
Baseline	13·0 (5·3); 120	13·4 (4·9); 115	12·8 (5·3); 119	..	..	..	..
10 weeks	12·1 (5·2); 89	11·8 (5·3); 91	11·8 (5·7); 95	0·05 (−1·08 to 1·19)	0·93	0·13 (−1·16 to 1·42)	0·84
28 weeks	10·6 (5·6); 78	11·0 (5·3); 87	11·7 (5·5); 83	−1·51 (−2·70 to −0·32)	0·013	−0·74 (−2·06 to 0·59)	0·28
56 weeks	11·6 (5·9); 75	10·8 (5·8); 89	12·9 (5·7); 81	−1·36 (−2·57 to −0·16)	0·027	−1·71 (−3·03 to −0·39)	0·011
**Work Productivity and Activity Impairment (overall work impairment)**							
Baseline	46·7 (26·8); 47	47·6 (26·0); 46	46·7 (25·0); 54	..	..	..	..
10 weeks	44·0 (25·4); 37	46·3 (27·4); 30	46·3 (27·1); 39	−3·82 (−13·80 to 6·16)	0·45	2·78 (−7·62 to 13·19)	0·60
28 weeks	38·0 (31·1); 33	46·5 (29·3); 29	40·7 (23·8); 33	−4·99 (−15·65 to 5·66)	0·36	5·19 (−5·63 to 16·02)	0·35
56 weeks	31·0 (21·6); 21	42·7 (23·9); 29	49·8 (25·0); 31	−15·58 (−27·41 to −3·74)	0·010	−4·01 (−15·08 to 7·05)	0·48
**Work Productivity and Activity Impairment (valued life activities)**							
Baseline	1·5 (0·8); 122	1·5 (0·8); 120	1·6 (0·8); 120				
10 weeks	1·3 (0·8); 90	1·4 (0·9); 93	1·5 (0·8); 94	−0·05 (−0·21 to 0·10)	0·50	0·04 (−0·11 to 0·19)	0·62
28 weeks	1·2 (0·8); 78	1·4 (0·9); 88	1·5 (0·9); 84	−0·21 (−0·37 to −0·05)	0·012	−0·01 (−0·17 to 0·15)	0·90
56 weeks	1·3 (0·9); 76	1·3 (0·9); 88	1·5 (0·9); 85	−0·18 (−0·35 to −0·02)	0·028	−0·08 (−0·24 to 0·08)	0·33

Data are shown as mean (SD); n, unless stated otherwise. A negative difference favours the intervention for all outcomes except Short Form-12 physical component summary and Short Form-12 mental component summary. CBA=cognitive behavioural approaches. HADS=Hospital Anxiety and Depression Scale. NRS=numerical rating scale. PEP=personalised exercise programmes.

* CIs are 95% CI except for Chalder Fatigue Scale and Fatigue Severity Scale, which are 97·5% CI.

The treatment effects on secondary outcomes were mixed at 56 weeks (table 2). Statistically significant effects were observed for both PEP and CBA on multidimensional fatigue scores, mental health-related quality of life, and sleep disturbance, and PEP additionally provided significant reductions in depression, increases in valued life activities, and reductions work disability. In particular, the effect on overall work impairment (Work Productivity and Activity Impairment −15·58 [95% CI −27·41 to −3·74], p=0·010) at 56 weeks was large. By contrast, neither treatment significantly improved pain, anxiety, or physical health-related quality of life (table 2). However, overall, both treatments improved general wellbeing. At 56 weeks, when asked how their global health status had changed since the start of the trial, 22 (24%) of 90 participants in the PEP group and 18 (17%) of 103 participants in the CBA group, compared with four (4%) of 102 participants in the usual care group, reported feeling either very much better or much better. In an exploratory analysis, PEP was superior to CBA with regards to global health status (p=0·0024; appendix p 14) and, overall, PEP consistently showed more positive effects than CBA for other outcomes, although these differences were not statistically significant (appendix pp 12–13).

We also did post-hoc subgroup analyses (appendix pp 16–17). First, differences in effects according to inflammatory rheumatic disease diagnosis were examined. At 56 weeks, participants with rheumatoid arthritis reported PEP effects on fatigue severity and impact similar to those in participants with an alternative inflammatory rheumatic disease diagnosis. At the same timepoint, participants with rheumatoid arthritis reported CBA reductions in fatigue that were superior to those in participants without rheumatoid arthritis, but both had similar reductions in fatigue impact. Second, although all participants had completed their scheduled treatment sessions before the onset of the COVID-19 pandemic UK lockdown (March 11, 2020), 124 (34%) remained under follow-up. Although this subgroup reported similar treatment effects in terms of fatigue impact, they reported less benefit in the terms of fatigue severity at 56 weeks (Chalder Fatigue Scale mean difference: PEP −2·68 [95% CI −6·19 to 0·84] and CBA −1·41 [–4·53 to 1·70]) than those who completed follow-up before pandemic lockdown (PEP −3·26 [–5·73 to −0·79] and CBA −2·84 [–5·20 to −0·47]). Gender-disaggregated data are reported in the appendix (pp 17–18).

A total of 425 adverse events were recorded, of which 61 (14%) were assessed as SAEs. The number of people who had at least one SAEs was similar across groups (12 participants in the PEP group, eight in the CBA group, and 14 in the usual care group) and no SAE was related to the trial (table 3). Of the 364 recorded adverse events (table 3), only one was related to the intervention (musculoskeletal trauma due to exercise).

**Table 3:** Safety outcomes of all study participants

		**PEP group (n=124)**	**CBA group (n=121)**	**Usual care group (n=122)**
Participants with at least one SAE		12 (10%)	8 (7%)	14 (11%)
Number of SAEs		17	19	25
SAEs criteria				
	SAEs requiring hospitalisation	13	17	20
	Medically significant SAEs	4	2	5
SAEs categories				
	Accident (including fractures and head injures)	1	1	2
	Cancer	1	2	2
	Cardiovascular disease	1	0	1
	Infection (severe)	0	2	6
	Inflammatory disease relapse (severe)	2	1	0
	Pregnancy or birth	0	0	2
	Surgery (including hospitalisation)	9	6	6
	Other	3	7	6
Participants with at least one adverse event		56 (45%)	62 (51%)	59 (48%)
Number of adverse events		136	117	111
Adverse event categories				
	Accident	10	11	14
	Cancer (suspected)	0	2	2
	Gastrointestinal	1	2	2
	Cardiovascular disease	6	5	1
	Inflammatory rheumatic disease flare-up	26	27	26
	Infection (bacterial, viral, or fungal; including SARS-CoV-2)	25	23	21
	Lightheaded or loss of consciousness	2	2	2
	Mental health	4	1	0
	Pain (including musculoskeletal-related pain)	14	11	9
	Respiratory	2	1	1
	Surgery (day case)	9	6	10
	Worsening of fatigue	1	0	1
	Other	36	26	22

Data are n or n (%) unless specified. CBA=cognitive-behavioural approaches. PEP=personalised exercise programmes. SAE=serious adverse event.

## Discussion

Our trial, the largest to evaluate fatigue-specific interventions for inflammatory rheumatic diseases, and the first to test remote delivery or generic approaches across heterogeneous diagnoses, found that PEP and CBA, when added to usual care, were safe and improved fatigue severity and impact among patients with a range of inflammatory rheumatic diseases compared with usual care alone. The benefits were maintained at 6 months after treatment completion. Additional benefits of improved mental health-related quality of life and sleep were observed for both interventions, and PEP also enhanced valued life activities and reduced levels of work disability and depression.

The effects of PEP and CBA were medium sized for the coprimary outcomes of fatigue severity and impact and more than the reported minimum clinically important reductions of the corresponding measures.^23^ The effects on secondary outcomes were generally small in magnitude, although it is likely that these might have cumulatively contributed to a clinically meaningful improvement in general wellbeing, as reflected by the important improvements in overall health status. In the context of the existing literature, these effects are favourable when compared with disease-specific interventions. Meta-analyses of physical activity and psychosocial interventions in rheumatoid arthritis report modest effects on fatigue reduction.^6^ Similarly, effects on fatigue have been reported in meta-analyses of biological immune therapies in rheumatoid arthritis^24^ and axial spondyloarthritis.^25^

Few trials for inflammatory rheumatic diseases have targeted fatigue as their primary outcome. In rheumatoid arthritis, small (n<100) fatigue-specific trials of physical activity have found improvements in fatigue;^26^ however, participants were not followed up after therapy completion. Physical activity adherence often declines after the completion of therapy and its benefits can be rapidly lost.^27^ The maintenance of PEP's effect at 56 weeks of follow-up is notable and could be explained by the integration of a behavioural component designed to disrupt unhelpful illness beliefs (eg, avoidance of fear), which might indirectly contribute to poor adherence. Enduring effects from psychosocial interventions are more consistently observed than physical interventions. The RAFT trial,^13^ which investigated reducing fatigue in rheumatoid arthritis, found a significant improvement in fatigue impact over 2 years. Similar to our CBA group, otherwise stable participants received a psychosocial intervention that was specific to fatigue, which was delivered by the rheumatology multidisciplinary team under specialist supervision and was compared with usual care. Although fatigue impact improved in the RAFT trial, fatigue severity did not. A direct comparison of the total Bristol Rheumatoid Arthritis Fatigue (BRAF) scale at 1 year, the only shared fatigue outcome measure between RAFT and LIFT, revealed similar fatigue reductions compared with CBA (RAFT CBA: mean difference −3·63; LIFT CBA: −4·86), but a less favourable fatigue reduction compared with that observed in PEP (mean difference for PEP −6·73).^13^ Moreover, RAFT was specific to rheumatoid arthritis and adopted face-to-face, group-based delivery. Although effectiveness might be reduced, there are cost benefits to group delivery. In the future, these interventions could be tested to assess the cost-effectiveness of hybrid individual and group remote delivery.

The benefits of remote delivery to enhance therapy accessibility are recognised and are especially attractive for this patient population given the fatiguing effects of travel. Furthermore, the COVID-19 pandemic has resulted in a rapid shift towards remote delivery across indications, often with inadequate evidence. Substantial supportive effectiveness data do exist for psychosocial interventions for mental health and interventions to promote physical activity that are delivered by telephone in the general population,^28^, ^29^ although long-term follow-up studies are limited in number. Our study is one of the few trials of remote delivery in inflammatory rheumatic disease, evidencing similar positive outcomes with reassuring improvements after 56 weeks of follow-up.

The mechanisms by which PEP and CBA exact their effects on fatigue are unknown. We do not anticipate that these interventions target the primary causes of fatigue (which remain uncharacterised), but hypothesise that they attenuate factors that maintain the persistence and impact of the symptom. For example, CBA aimed to replace unhelpful beliefs and behaviours that can exacerbate fatigue. This more focused approach might explain why only specific fatigue domains (ie, physical and emotional domains of fatigue as measured by the BRAF) improved among CBA participants. In contrast, the established pleiotropic effects of physical activity are likely to explain the pan-domain fatigue improvements observed in PEP (appendix p 15).

LIFT was primarily designed to be a pragmatic trial. Its major strength is its generalisability to a typical rheumatology service and its selection of a sizeable, but often overlooked, group of patients who report chronic fatigue despite adequate management of their inflammation. Inflammation is one of many factors that contribute to inflammatory rheumatic disease-related fatigue and, in real-world practice, rheumatologists prioritise the treatment of inflammation in the first instance before considering alternative approaches for fatigue. External validity was enhanced by embedding the trial within several rheumatology services. The interventions were delivered by members of the multidisciplinary teams who integrated their therapist duties within their standard clinical schedules and this study indicates that psychological and physical therapy skills can be efficiently acquired by relevant health professionals in rheumatology. In doing so, the trial was susceptible to the standard challenges faced by health-care services—eg, waiting lists and staff turnover due to illness and changing roles.

Despite our trial being methodologically rigorous, several limitations exist. First, full masking was not possible due to the need to engage people in behavioural change. Moreover, the comparator was treatment as usual (usual care) since the intention of our trial was to assess whether our interventions improved on current practice. The potential for resultant detection bias was mitigated by masking investigators and analysts to allocation. Non-specific treatment effects, such as placebo, exist in real-world practice; however, we aimed to minimise such effects by designating our primary endpoint at 56 weeks, 6 months after therapy. Also, the risk of nocebo effects in relation to our comparator did not appear substantial. As a minimum, participants in the usual care group received established educational materials, which have previously been associated with a positive impact^30^ and, within this trial, were related to improved outcomes compared with the baseline score and equivalent attrition rates. Second, 12% of participants given PEP and CBA discontinued their respective therapies due to multiple reasons. However, these rates were in line with our previous data^31^ and 53% of these patients still contributed to the primary outcome. Third, we were unable to fully assess whether or not intervention participants adapted or implemented what was being prescribed.

The issue of missing outcome data should be framed within the context of the COVID-19 pandemic. Although 33% of participants remained under follow-up at pandemic onset, our capacity to capture several outcomes remotely enabled 77% of all primary outcomes to be recorded at 56 weeks, close to our a priori 80% follow-up estimate. The pandemic might also have biased the effects of the interventions. The majority of this patient population would have been classed as clinically vulnerable and advised to isolate in their homes, possibly compromising some of the core self-management aspects of CBA and PEP (eg, physical activity). Indeed, our post-hoc subgroup analysis indicates decreased PEP and CBA effects on fatigue severity among these participants. Thus, without these extraordinary pandemic conditions, the benefits of both interventions might have been even larger. We chose not to define adherence according to session attendance due to anticipated wide variation in individual participant needs. In fact, the complier average causal effect analysis supports this decision for CBA; however, it seems that superior PEP outcomes are reached if participants attend at least three sessions, and so a minimum attendance should be prescribed in future practice. Finally, this trial was not powered to examine consistency of intervention effects across specific inflammatory rheumatic diseases. Consistent with routine care, the trial population included several inflammatory rheumatic diseases of varying prevalence, the most common being rheumatoid arthritis. Although the size of effects of PEP were similar in our post-hoc subgroup analysis of participants with rheumatoid arthritis and without rheumatoid arthritis, participants with rheumatoid arthritis appeared to have larger CBA effects compared with those without rheumatoid arthritis. One potential explanation is that CBA was originally informed by a rheumatoid arthritis-specific psychosocial intervention.

In the UK and elsewhere, there are currently no formally recommended treatments specific to fatigue for patients with inflammatory rheumatic diseases. By supporting the prescription of expensive immune therapeutics, health-care providers have afforded independence from physical disability to a new generation of patients. By alleviating the ongoing and invisible disability of fatigue, patients can have a fuller independence, which, in turn, will enable health-care providers to maximise the gains of their investment in immune therapeutics. Our results now support the prescription of both PEP and CBA for inflammatory rheumatic disease-related fatigue. These are not the first non-pharmacological interventions to be successfully tested for fatigue; however, in practice, few rheumatology services provide evidence-based, fatigue-specific therapies due to implementation challenges. The data presented in this Article offer robust evidence to overcome existing implementation barriers and subsequently enable widespread access. The versatility of remote delivery is especially timely in the context of the COVID-19 pandemic, allowing both patients and therapists to interact from the safety of their homes. Moreover, the remote model offers an efficient opportunity towards centralising health-care service provision across multiple sites and regions, and delivery by the rheumatology multidisciplinary teams, rather than sparsely available specialists, will enhance accessibility. Finally, these data support a standardised approach to fatigue management across the spectrum of inflammatory rheumatic diseases, eliminating the operational challenges of disease-specific programmes and ensuring inclusivity of care. Fatigue is a patient priority across the spectrum of chronic disease. The transdiagnostic benefits of CBA and, especially, PEP in inflammatory rheumatic disease would support their testing in other clinical populations. However, although at least as comparable to other fatigue interventions, the effects of PEP and CBA were moderate in size with substantial numbers of patients continued to report clinically-relevant fatigue. Additionally, it is unknown whether these effects will be maintained beyond 1 year. In the future, effects might be optimised by targeting those patients most likely to receive a larger benefit from either PEP or CBA, integrating clinical and biological markers to derive useful clinical decision tools or applying a combined PEP and CBA approach. Moreover, booster sessions might be required to prolong their benefits longer term.

In conclusion, CBA and PEP delivered by telephone provided statistically and clinically significant reductions in fatigue severity and impact for a wide range of patients whose disease was otherwise stable. The treatments were well tolerated, their benefits were maintained 6 months after treatment completion, and they were successfully delivered by members of the rheumatology multidisciplinary teams after specialist training.

## Data sharing

Anonymised individual patient data will be made available following any reasonable request made to the corresponding author, subject to a data sharing agreement and UK research governance regulations. The intervention manuals can be found on https://www.abdn.ac.uk/iahs/research/epidemiology/lift-1286.php.

## Declaration of interests

We declare no competing interests.

## References

[1] Crowson CS, Matteson EL, Myasoedova E (2011). The lifetime risk of adult-onset rheumatoid arthritis and other inflammatory autoimmune rheumatic diseases. Arthritis Rheum.

[2] Smith E, Hoy DG, Cross M (2014). The global burden of other musculoskeletal disorders: estimates from the Global Burden of Disease 2010 study. Ann Rheum Dis.

[3] Repping-Wuts H, van Riel P, van Achterberg T (2009). Fatigue in patients with rheumatoid arthritis: what is known and what is needed. Rheumatology (Oxford).

[4] van Hoogmoed D, Fransen J, Bleijenberg G, van Riel P (2010). Physical and psychosocial correlates of severe fatigue in rheumatoid arthritis. Rheumatology (Oxford).

[5] Dures E, Cramp F, Hackett K, Primdahl J (2020). Fatigue in inflammatory arthritis. Best Pract Res Clin Rheumatol.

[6] Cramp F, Hewlett S, Almeida C (2013). Non-pharmacological interventions for fatigue in rheumatoid arthritis. Cochrane Database Syst Rev.

[7] Hulme K, Safari R, Thomas S (2018). Fatigue interventions in long term, physical health conditions: a scoping review of systematic reviews. PLoS One.

[8] Dures E, Almeida C, Caesley J (2014). A survey of psychological support provision for people with inflammatory arthritis in secondary care in England. Musculoskelet Care.

[9] Brenes GA, Ingram CW, Danhauer SC (2011). Benefits and challenges of conducting psychotherapy by telephone. Prof Psychol Res Pr.

[10] Martin KR, Bachmair EM, Aucott L (2019). Protocol for a multicentre randomised controlled parallel-group trial to compare the effectiveness of remotely delivered cognitive-behavioural and graded exercise interventions with usual care alone to lessen the impact of fatigue in inflammatory rheumatic diseases (LIFT). BMJ Open.

[11] Zigmond AS, Snaith RP (1983). The Hospital Anxiety and Depression Scale. Acta Psychiatr Scand.

[12] White PD, Goldsmith KA, Johnson AL (2011). Comparison of adaptive pacing therapy, cognitive behaviour therapy, graded exercise therapy, and specialist medical care for chronic fatigue syndrome (PACE): a randomised trial. Lancet.

[13] Hewlett S, Almeida C, Ambler N (2019). Group cognitive-behavioural programme to reduce the impact of rheumatoid arthritis fatigue: the RAFT RCT with economic and qualitative evaluations. Health Technol Assess.

[14] Cella M, Chalder T (2010). Measuring fatigue in clinical and community settings. J Psychosom Res.

[15] Krupp LB, LaRocca NG, Muir-Nash J, Steinberg AD (1989). The Fatigue Severity Scale. Application to patients with multiple sclerosis and systemic lupus erythematosus. Arch Neurol.

[16] Nicklin J, Cramp F, Kirwan J, Greenwood R, Urban M, Hewlett S (2010). Measuring fatigue in rheumatoid arthritis: a cross-sectional study to evaluate the Bristol rheumatoid arthritis fatigue multi-dimensional questionnaire, visual analog scales, and numerical rating scales. Arthritis Care Res (Hoboken).

[17] Ware J, Kosinski M, Keller SDA (1996). A 12-item short-form health survey: construction of scales and preliminary tests of reliability and validity. Med Care.

[18] McCaffery M, Beebe A (1994).

[19] Jenkins CD, Stanton BA, Niemcryk SJ, Rose RM (1988). A scale for the estimation of sleep problems in clinical research. J Clin Epidemiol.

[20] Reilly MC, Zbrozek AS, Dukes EM (1993). The validity and reproducibility of a Work Productivity and Activity Impairment instrument. PharmacoEconomics.

[21] Katz PP, Radvanski DC, Allen D (2011). Development and validation of a short form of the valued life activities disability questionnaire for rheumatoid arthritis. Arthritis Care Res (Hoboken).

[22] Norman GR, Sloan JA, Wyrwich KW (2003). Interpretation of changes in health-related quality of life: the remarkable universality of half a standard deviation. Med Care.

[23] Nordin Å, Taft C, Lundgren-Nilsson Å, Dencker A (2016). Minimal important differences for fatigue patient reported outcome measures—a systematic review. BMC Med Res Methodol.

[24] Almeida C, Choy EH, Hewlett S (2016). Biologic interventions for fatigue in rheumatoid arthritis. Cochrane Database Syst Rev.

[25] Shim J, Dean LE, Karabayas M, Jones GT, Macfarlane GJ, Basu N (2020). Quantifying and predicting the effect of anti-TNF therapy on axSpA-related fatigue: results from the BSRBR-AS registry and meta-analysis. Rheumatology (Oxford).

[26] Katz P, Margaretten M, Gregorich S, Trupin L (2018). Physical activity to reduce fatigue in rheumatoid arthritis: a randomized controlled trial. Arthritis Care Res (Hoboken).

[27] Marcus BH, Dubbert PM, Forsyth LH (2000). Physical activity behavior change: issues in adoption and maintenance. Health Psychol.

[28] Efron G, Wootton BM (2021). Remote cognitive behavioral therapy for panic disorder: a meta-analysis. J Anxiety Disord.

[29] Foster C, Richards J, Thorogood M, Hillsdon M (2013). Remote and web 2.0 interventions for promoting physical activity. Cochrane Database Syst Rev.

[30] Hart RI, Ng WF, Newton JL, Hackett KL, Lee RP, Thompson B (2017). What impact does written information about fatigue have on patients with autoimmune rheumatic diseases? Findings from a qualitative study. Musculoskelet Care.

[31] McBeth J, Prescott G, Scotland G (2012). Cognitive behavior therapy, exercise, or both for treating chronic widespread pain. Arch Intern Med.

