# Supplementary material for: Remotely delivered cognitive behavioural and personalised exercise interventions for fatigue severity and impact in inflammatory rheumatic diseases (LIFT): a multicentre, randomised, controlled, open-label, parallel-group trial
Source: Lancet Rheumatol. 2022 Jun 27;4(8):e534–45. doi: 10.1016/S2665-9913(22)00156-4 (PMC9646481; doi:10.1016/S2665-9913(22)00156-4)
Supplement: Supplementary appendix [file mmc1.pdf]

THE LANCET
Rheumatology

Supplementary appendix

This appendix formed part of the original submission and has been peer reviewed. We post it as supplied by the authors.

Supplement to: Bachmair E-M, Martin K, Aucott L, et al. Remotely delivered cognitive behavioural and personalised exercise interventions for fatigue severity and impact in inflammatory rheumatic diseases (LIFT): a multicentre, randomised, controlled, open-label, parallel-group trial. *Lancet Rheumatol* 2022; published online June 27. https://doi.org/10.1016/S2665-9913(22)00156-4.

Appendix

Table of contents

Methods 2
Table 1 Full list of inclusion and exclusion criteria 2
LIFT study group 2
TIDieR check lists for interventions 3
Supplementary description of statistical methods 9
Results 10
Table 2 Reasons for ineligibility 10
Table 3 Demographics of visit 4 responders (those who either withdraw consent or were loss to follow-up) compared to non-responders 10
Table 4 Multiple imputation: Chalder Fatigue Scale 11
Table 5 Multiple Imputation: Fatigue Severity Scale 11
Table 6 Comparison PEP vs CBA for primary and secondary outcomes 12
Table 7 Global Health status outcome and comparison of interventions 14
Additional post-hoc analyses 16
Effect of compliance 16
Table 9 Primary Outcomes summaries and CACE Models estimates to incorporating the effect of compliance 16
Sub-group analyses 16
Table 10 Post hoc Sub-Group analyses for Disease Type impact: Chalder Fatigue Scale 16
Table 11 Post hoc Sub-Group analyses for Disease type: Fatigue Severity Scale 17
Covid impact 17
Table 12 Post hoc Sub-Group analyses for COVID impact: Chalder Fatigue Scale 17
Table 13 Post hoc Sub-Group analyses COVID impact: Fatigue Severity Scale 17
Table 14 Post hoc Sub-Group analyses for Gender impact: Chalder Fatigue Scale 18
Table 15 Post hoc Sub-Group analyses for Gender impact: Fatigue Severity Scale 18
Table 16 Training and Supervision time 18
Table 17 Trial amendments 18

Methods

Table 1 Full list of inclusion and exclusion criteria

**In order to be considered eligible for participation in the study they must:**	
Criterion	Characteristics of eligible participants
1.	Be male or female aged ≥ 18 years at the time of consent
2.	Have been diagnosed with an IRD such as RA, SLE or AxSpA by a consultant rheumatologist
3.	Report fatigue to be a persistent problem as evidenced by answering both questions:<br>1. Have you had problems with fatigue for more than three months? (Yes)<br>2. Please circle the number that shows your average level of fatigue during the past 7 days. (≥ 6 based on a numerical rating scale of 0 (no fatigue) to 10 (totally exhausted))
4.	Have access to a telephone landline or mobile telephone and/or internet based audio/video calls
5.	Give permission for researchers to access their hospital medical notes
6.	Have stable disease as evidenced by no change in immunomodulatory therapy within the last three months based on hospital medical records
7.	Currently be under the care of a secondary care physician
**Participants will be excluded if:**	
Criterion	Characteristics of ineligible participants
1.	there are significant abnormalities in thyroid function (TSH levels) in the most recent blood test done within the last three months
2.	there is evidence of severe anaemia (haemoglobin levels) in the most recent blood test done within the last three months
3.	there is evidence of severe renal dysfunction (eGFR) in the most recent blood test done within the last three months
4.	they have a medical condition which would make the proposed interventions unsuitable, e.g. significant heart disease
5.	they are pregnant
6.	they are unable to understand English sufficiently to take part in the intervention
7.	they are unable to provide written informed consent
8.	they are not willing to be randomised
9.	they are currently participating in an interventional clinical trial

Abbreviations: AxSpA, Axial Spondyloarthritis; eGFR, estimated glomerular filtration rate; RA, rheumatoid arthritis; SLE, Systemic Lupus Erythematosus, TSH, Thyroid Stimulating Hormone

LIFT study group

Amy Nicol, Karen Norris, Sandra Mann, Lorna Van Lierop, Eli Gomez, Fiona McCurdy, Valerie Findlay, Neil Hastie, Eunice Morgan, Roselyn Emmanuel, Daniel Whibley, Aimee Urquart, Laura MacPerson (NHS Grampian, UK); Janice Rowland, Gwen Kiddie, Debbie Pankhurst, Paul Johnstone, Hilary Nicholson, Angela Dunsmore, Alison Knight, John Ellis, Callum Maclean, Linda Crighton, Cameron Shearer (NHS Tayside, UK);
Judy Coyle, Susan Begg, Lyndsey Ackerman, Jill Carnevale, Samantha Arbuthnot, Helen Watters, Dervil Dockrell, Debbie Hamilton (NHS Lothian, UK); Dario Salutous, Susanne Cathcart, Dominic Rimmer, Emma Hughes, Juliet Harvey, Mairi Gillies, Susan Webster, Leeanne Milne, Gary Semple, Katharine Duffy, Lynne Turner, John Alexander, June Innes, Charlotte Clark, Christine Meek, Elizabeth McKenna (NHS Greater Glasgow & Clyde, UK); Christine Routledge, Helain Hinchcliffe-Hume, Emmanuella Traianos, Beth Dibnah, David Storey, Gemma O'Callaghan, Jenny Yael Baron, Sally Hunt (Newcastle upon Tyne NHS Trust); Natalie Wheat, Pam Smith, Elizabeth Ann Barcroft, Amy Thompson, Johanne Tomlinson (Haywood Hospital, Stoke on Trent, UK); Jill Barber, Gladys MacPerson (University of Aberdeen, UK); Peter White (Queen Mary University of London, UK); Sarah Hewlett (University of the West of England, Bristol, UK).

TIDieR check lists for interventions

The TIDieR (Template for Intervention Description and Replication) Checklist:

Item number	Item	Primary paper (page or appendix number)	Other (details)
	**BRIEF NAME**		
**1.**	Provide the name or a phrase that describes the intervention.	Personalised Exercise Programme (PEP)	______________
	**WHY**		
**2.**	Describe any rationale, theory, or goal of the elements essential to the intervention.	PEP assumes that IRDs are associated with deconditioning, reduced physical strength and altered perception of effort consequent upon reduced physical activity. These changes are thought to be reversible, and thus improving fitness and physical functioning will alter perception of effort, enable the body to gain fitness and strength, leading to a reduction in symptoms and an increase in activity capacity.<br><br>There may be other mechanisms involved in the success of PEP apart from reversing deconditioning, including elements of habituation, and positive effects of re-engagement with important activities. Participants were encouraged, in an individualised, symptom limited manner, to gradually alter their physical activity pattern with the aim of achieving the national physical activity guidelines, of 150 minutes of moderate intensity exercise/physical activity (60-75% of age related maximum heart rate) per week. Moderate intensity exercise/physical activity is required to improve physical fitness.<br><br>Evidenced based behavioural change techniques such as goal setting, action planning, social support and feedback were employed, which also helped optimise adherence.	_____________
	**WHAT**		
**3.**	Materials: Describe any physical or informational materials used in the intervention, including those provided to participants or used in intervention delivery or in training of intervention providers. Provide information on where the materials can be accessed (e.g. online appendix, URL).	Therapists were provided with a fully referenced manual, available at the study website, which outlined the rationale for, the components of, and the techniques used to deliver the eight sessions of the intervention.<br>Participants were provided with a comprehensive manual containing all the material they required for the intervention<br>(available at the study website).	_____________

		Therapists were trained to deliver the PEP intervention by experienced researchers (a senior physiotherapist and a senior exercise scientist), in groups of 2-6, over 1 day, using a mixture of short lectures, demonstrations, case studies and discussions. Training materials are available at the study website. Telephone supervision was provided by the trainers to each therapist on an individual basis, for about 30 minutes every 4 weeks.<br><br>Study website: https://www.abdn.ac.uk/iahs/research/epidemiology/bsrbras-1286.php	
**4.**	Procedures: Describe each of the procedures, activities, and/or processes used in the intervention, including any enabling or support activities.	The intervention was personalised to address participants particular problems but core components were formation of a problem statement, completion of activity diaries, goal setting, homework activities, review and feedback.	______________
	**WHO PROVIDED**		
**5.**	For each category of intervention provider (e.g. psychologist, nursing assistant), describe their expertise, background and any specific training given.	Therapists delivering the PEP were senior NHS physiotherapy member(s) of the participant's local standard care multi-disciplinary team. For local teams where more than one therapist was trained to deliver PEP, participants were pragmatically allocated to the therapist with the greatest capacity, in the context of their NHS role, at the time of randomisation.	______________
	**HOW**		
**6.**	Describe the modes of delivery (e.g. face-to-face or by some other mechanism, such as internet or telephone) of the intervention and whether it was provided individually or in a group.	The initial PEP session was conducted face to face and all subsequent sessions were provided to individual participants via pre-arranged telephone calls.	______________
	**WHERE**		
**7.**	Describe the type(s) of location(s) where the intervention occurred, including any necessary infrastructure or relevant features.	Participants were recruited from specialist rheumatology clinics. They received their first face to face session in a local NHS out-patient physiotherapy department or rheumatology clinic and received the remaining intervention (therapist telephone calls) in their own homes.	______________
	**WHEN and HOW MUCH**		
**8.**	Describe the number of times the intervention was delivered and over what period of time including the number of sessions, their schedule, and their duration, intensity or dose.	The PEP intervention consisted of one face to face session, delivered in week 0, and 7 telephone sessions, delivered in weeks 1,2,4,6,10,14 and 22. Session 1 lasted approximately 1 hour, subsequent sessions were around 30 minutes.	______________
	**TAILORING**		
**9.**	If the intervention was planned to be personalised, titrated or adapted, then describe what, why, when, and how.	All participants were scheduled to receive the full eight sessions. Participants completed detailed physical activity diaries in weeks 1 and 2. Participants also set personalised goals in terms of what they wanted to achieve from the programme. The baseline data from the	______________

		diaries, together with the goals were used to plan the personalised progressive exercise programme, which was agreed between participant and therapist.<br>The strengthening exercise component of the physical activity guidelines were only considered if deemed appropriate by the therapist.	
	**MODIFICATIONS**		
**10.**[ǂ]	If the intervention was modified during the course of the study, describe the changes (what, why, when, and how).	The intervention as described in the therapists' manual was not modified during the course of the intervention.	______________
	**HOW WELL**		
**11.**	Planned: If intervention adherence or fidelity was assessed, describe how and by whom, and if any strategies were used to maintain or improve fidelity, describe them.	Therapists were requested to audio-record X 5% (n=44) randomly selected therapy sessions using an algorithm that takes into account session number, therapist, site location, patient gender. However, this was subject to participant agreeing to be recorded and treatment adherence (no sessions completed). Tapes were listened to by the supervisors and discussed during supervision sessions.	______________
**12.**[ǂ]	Actual: If intervention adherence or fidelity was assessed, describe the extent to which the intervention was delivered as planned.	N/A	______________

The TIDieR (Template for Intervention Description and Replication) Checklist:

TIDieR
Template for Intervention Description and Replication

Item number	Item	Primary paper (page or appendix number)	Other
	**BRIEF NAME**		
**1.**	Provide the name or a phrase that describes the intervention.	Cognitive behavioural approach (CBA)	______________
	**WHY**		
**2.**	Describe any rationale, theory, or goal of the elements essential to the intervention.	The CBA suggests that while fatigue may be precipitated by disease related factors, it is maintained by multiple interacting cognitive, behavioural, emotional and biological factors. Changing the way that people think about, respond to, and feel about their fatigue can reduce fatigue or make it more manageable. Participants were introduced to a simple model showing how the above-mentioned factors can interact, were invited to develop a personalised problem statement that described their own fatigue in terms of the model, and were encouraged to form relevant and feasible goals and action plans to address their fatigue. Therapists used techniques such as behavioural experiments, problem solving and cognitive restructuring to help patients to bring about changes to their behaviour, thinking and emotions. Progress was reviewed at each session, feedback provided, and new goals set if appropriate.<br><br>Goal setting, feedback and action planning were adopted to enhance adherence.	_____________
	**WHAT**		
**3.**	Materials: Describe any physical or informational materials used in the intervention, including those provided to participants or used in intervention delivery or in training of intervention providers. Provide information on where the materials can be accessed (e.g. online appendix, URL).	Therapists were provided with a fully referenced manual, available at the study website, which outlined the rationale for, the components of, and the techniques used to deliver the eight sessions of the intervention. Patients were provided with 10 handouts, available at the study website, for use during the intervention; these were included as an appendix in the therapists' handbook. Therapists were trained to deliver the CBA intervention by two experienced researchers (a mental health specialist and a health psychologist), in groups of 2-6, over 1 or 2 days, using a mixture of short lectures, demonstrations, quizzes, discussions,	_____________

		and role plays. Training materials are available at the study website. Telephone supervision was provided by the trainers to each therapist on an individual basis, for about 30 minutes every 2-4 weeks.<br><br>Study website: https://www.abdn.ac.uk/iahs/research/epidemiology/bsrbras-1286.php	
**4.**	Procedures: Describe each of the procedures, activities, and/or processes used in the intervention, including any enabling or support activities.	The intervention was personalised to address participants particular problems but core components were formation of a problem statement, completion of activity diaries, goal setting, homework activities, review and feedback.	______________
	**WHO PROVIDED**		
**5.**	For each category of intervention provider (e.g. psychologist, nursing assistant), describe their expertise, background and any specific training given.	Therapists were health care professionals with clinical experience of working with patients with inflammatory rheumatoid diseases in the participant's local multi-disciplinary team. Their professional backgrounds were specialist rheumatology nurse (n=2); occupational therapist (n=8); speciality research nurse (n=1). For local teams where more than one therapist was trained to deliver CBA, participants were pragmatically allocated to the therapist with the greatest capacity, in the context of their NHS role, at the time of randomisation	______________
	**HOW**		
**6.**	Describe the modes of delivery (e.g. face-to-face or by some other mechanism, such as internet or telephone) of the intervention and whether it was provided individually or in a group.	All CBA therapy sessions were provided to individual participants via pre-arranged telephone calls.	______________
	**WHERE**		
**7.**	Describe the type(s) of location(s) where the intervention occurred, including any necessary infrastructure or relevant features.	Participants were recruited from specialist rheumatology clinics, and received the intervention (therapist telephone calls) in their own homes.	______________
	**WHEN and HOW MUCH**		
**8.**	Describe the number of times the intervention was delivered and over what period of time including the number of sessions, their schedule, and their duration, intensity or dose.	The CBA intervention consisted of 8 telephone sessions, delivered in weeks 0,1,2,4,6,10,14 and 22. Sessions 1 and 2 lasted approximately 1 hour, subsequent sessions were 30-45 minutes.	______________
	**TAILORING**		
**9.**	If the intervention was planned to be personalised, titrated or adapted, then describe what, why, when, and how.	All participants were scheduled to receive the full eight sessions. Because the problem statement was personalised, priorities for change and goals set were also personalised.	______________

		Participants were free to use the 10 handouts provided to support their activities and completion of homework between session, but not all participants used all the handouts. For example, if sleep patterns were not a problem, the handout on sleep may not have been used.	
	**MODIFICATIONS**		
**10.**[ǂ]	If the intervention was modified during the course of the study, describe the changes (what, why, when, and how).	The intervention as described in the therapists' manual was not modified during the course of the intervention.	______________
	**HOW WELL**		
**11.**	Planned: If intervention adherence or fidelity was assessed, describe how and by whom, and if any strategies were used to maintain or improve fidelity, describe them.	Therapists were requested to audio-record 5% (n=44) randomly selected therapy sessions using an algorithm that takes into account session number, therapist, site location, patient gender. However, this was subject to participant agreeing to be recorded and treatment adherence (no sessions completed). Tapes were listened to by the supervisors and discussed during supervision sessions.	______________
**12.**[ǂ]	Actual: If intervention adherence or fidelity was assessed, describe the extent to which the intervention was delivered as planned.	N/A	______________

Supplementary description of statistical methods

1) *Main treatment comparison method analyses – primary and secondary variables*

Outcomes were analysed using a heteroscedastic partially nested repeated measures mixed effects linear model. This model included the baseline version of the score, and binary fixed effects variables for (nominal time) scoring >10 on the HADS depression subscale. Treatment effects were estimated from the treatment-by-time interaction, the main time point of interest was 56 weeks. A random effect for therapist was included in the CBA arm only to incorporate clustering due to therapist, there was no evidence of therapist effect in the PEP arm, a random effect for centre was included for the PEP and control group. Degrees of freedom were adjusted for the small number of clusters using the Kenward Rogers method. The primary approach used all follow-up data and analysed-as-randomised approach under a missing-at-random assumption, a modified intention-intention-to-treat analysis. The only outcome not assessed in this way was change in Global Health status, an ordinal variable and treated as such using a generalised linear model with a logit link and specified as being ordinal. The ordered logit model with therapist included would not converge, so this model only includes centre.

Sensitivity analyses:

Various sensitivities were conducted but only on the Primary Outcome(s), CF and FSS.

a) A Complier Average Causal Effect (CACE) analysis was conducted for the primary outcomes as a sensitivity analysis. Compliance was defined as attendance of 3 or more sessions. The analyses were conducted fitting linear models where compliance was endogenously determined in this case via a two-stage least squares model. This was done for each active arm (PEP and CBA) separately relative to UC with the same adjustments as the main analyses but only at 56 weeks. This model accounted for clusters using a robust variance rather than by random effects as in the main models, due to convergence issues.

b) Primary outcomes underwent multiple imputation. We considered 6 scenarios for missingness imputation:

Multiple imputation scenarios

**Active:All base; UC:All MICE**	If missing and in an active arm (PEP or CBA), baseline values were imputed; if UC arm MI generated were imputed (see **100% MICE below**): this detriments active arms compared to some change allowed for UC
**All base**	If missing, impute the baseline value: no change over time allowed but for all arms
**50% base:50% MICE**	If missing then impute 50% (see **100% MICE below**) and 50% using the baseline value.
**33.3% base:66.7% MICE**	If missing then impute 33.3% (see **100% MICE below**) and 66.7% using the baseline value.
**25% base:75% MICE**	If missing then impute 25% (see **100% MICE below**) and 75% using the baseline value.
**100% MICE**	Missing values were imputed using chained equations incorporating the basic model adjustment variables along with age gender and others deemed relevant to that outcome. This approach assumes the outcome to be missing at random. Multiple imputations were done for each treatment arm separately then combined.

All models used multiple imputation data sets. These were adjusted for their baseline outcome measure, HADS depression subscale >10 at baseline as fixed effects fixed effect with Centre clustering and individuals nested within centre's as random effects.

For all scenarios, an appropriate number of simulated data sets were imputed according to the proportion missing and combined using Rubin's rule for use in the original outcome model form to give another estimate of the treatment comparison effects for affected outcomes.

c) Diagnosis disease type Sub-group analysis: Participants with Rheumatoid arthritis (RA), Spondylitis (SpA),) Connective tissue disease (CTD) or other at baseline were compared by incorporating an interactive term to denote those with each of these disease types. These were all developed within one model but have been reported separately for ease of interpretation.

d) The impact of COVID was investigated by flagging those whose follow-up was completed after the 11/03/20 (prior to the UK official lockdown but when re-arrangements were started for continuing data collection as far as possible through this period). This dichotomous approach was a crude but harsh attempt. Again, treatments were compared but this time incorporating an interactive term to denote those with effected by Covid at all and those with completed follow-up prior to lockdown. Again, these were reported separately although generated within the same model

Results

Reasons for ineligibility

Table 2.1 **Reasons for being ineligible- not mutually exclusive (n=866)**

Fatigue less than 6/ No Fatigue in last 3 Months	370
Other not eligible	307
Some other condition	120
Didn't want to	66
Refused Permission for further contact	59
Immunomodulatory therapy recently changed	55
Permission to medical notes denied	54
No Response to calls	41
Not recently seen by Rheumatologist	31
Recent admission	24
Unable to exercise	19
Not under secondary care	7
Part of another intervention	7
Joint replacement in last 3 months	4
No English	2
Pregnant	2
Could not confirm eligible	2
Joint replacement 3-6 months ago but Dr not approved	1

Table 2.2 **Reasons for people who consented but by randomisation were ineligible- not mutually exclusive (n=10)**

Fatigue Average <=5	6
BP>200	2
Bloods not collectable and no values in medical notes.	2

Table 3 Demographics of visit 4 responders (those who either withdraw consent or were loss to follow-up) compared to non-responders

**Variable**	**Responders (295)**	**Non-Responders (72)**
**Age [years]#**	57.94 (12.17): 295	55.69 (14.67: 71
**Gender†**		
Female	222 (75.3)	52 (72.2)
Male	73 (24.7)	19 (26.4)
Missing	. (.)	1 (1.4)
**Marital status†**		
Single	30 (10.2)	10 (13.9)
Married	189 (64.1)	39 (54.2)
Widowed	20 (6.8)	5 (6.9)
Divorced	21 (7.1)	8 (11.1)
Separated	11 (3.7)	2 (2.8)
Living with partner or spouse	23 (7.8)	4 (5.6)
Missing	1 (0.3)	4 (5.6)
**Employment Group†**		
Working full-time (30+hrs /week)	81 (27.5)	28 (38.9)
Working part-time (<30+hrs /week)	49 (16.6)	6 (8.3)
Unemployed and looking for work	4 (1.4)	. (.)
Unable to work because of illness or disability	41 (13.9)	9 (12.5)
At home and not looking for paid employment	8 (2.7)	1 (1.4)
Student	2 (0.7)	3 (4.2)
Retired	102 (34.6)	22 (30.6)
Other	7 (2.4)	. (.)
missing	1 (0.3)	3 (4.2)
**Education level†**		
Secondary school	86 (29.2)	19 (26.4)
Apprenticeship	12 (4.1)	3 (4.2)
Further education college	94 (31.9)	26 (36.1)
University degree	68 (23.1)	16 (22.2)
Further degree	31 (10.5)	4 (5.6)
missing	4 (1.4)	4 (5.6)
**Ethnic Group†**		
Scottish	215 (72.9)	56 (77.8)
Other British	64 (21.7)	9 (12.5)

Irish	1 (0.3)	. (.)
Other White	10 (3.4)	3 (4.2)
Other Ethnic	1 (0.3)	. (.)
missing	4 (1.4)	4 (5.6)

#Continuous data: mean (sd); n

†Categorical data: N n(%)

Table 4 Multiple imputation: Chalder Fatigue Scale

	**PEP vs UC**		**CBA vs UC**		**PEP vs CBA**	
	**Estimate**	**p value**	**Estimate**	**p value**	**Estimate**	**p value**
**Active:All base; TAU:All MICE**						
10 weeks	-0.86 (-2.31 to 0.59)	0.246	-0.27 (-1.73 to 1.19)	0.716	-0.59 (-2.01 to 0.83)	0.417
28 weeks	-1.81 (-3.32 to -0.30)	0.019	-1.59 (-3.11 to -0.07)	0.040	-0.22 (-1.64 to 1.20)	0.762
56 weeks	-1.53 (-3.01 to -0.05)	0.043	-1.76 (-3.25 to -0.27)	0.020	0.23 (-1.19 to 1.65)	0.750
**All base**						
10 weeks	-1.26 (-2.66 to 0.13)	0.075	-0.65 (-2.05 to 0.75)	0.364	-0.61 (-2.01 to 0.78)	0.389
28 weeks	-2.42 (-3.81 to -1.02)	0.001	-2.17 (-3.57 to -0.77)	0.002	-0.25 (-1.64 to 1.15)	0.729
56 weeks	-1.99 (-3.38 to -0.59)	0.005	-2.19 (-3.59 to -0.79)	0.002	0.20 (-1.19 to 1.60)	0.775
**50% base:50% MICE**						
10 weeks	-1.50 (-2.98 to -0.02)	0.047	-0.71 (-2.22 to 0.79)	0.352	-0.79 (-2.31 to 0.74)	0.312
28 weeks	-2.94 (-4.55 to -1.34)	<0.001	-2.33 (-3.86 to -0.79)	0.003	-0.62 (-2.28 to 1.05)	0.468
56 weeks	-2.75 (-4.30 to -1.20)	0.001	-2.28 (-3.77 to -0.79)	0.003	-0.47 (-2.02 to 1.08)	0.551
**33.3% base:66.7% MICE**						
10 weeks	-1.56 (-3.07 to -0.04)	0.044	-0.72 (-2.26 to 0.82)	0.358	-0.84 (-2.41 to 0.74)	0.299
28 weeks	-3.25 (-4.79 to -1.70)	<0.001	-2.41 (-3.98 to -0.83)	0.003	-0.84 (-2.45 to 0.77)	0.308
56 weeks	-2.97 (-4.57 to -1.37)	<0.001	-2.33 (-3.85 to -0.80)	0.003	-0.64 (-2.21 to 0.92)	0.420
**25% base:75% MICE**						
10 weeks	-1.60 (-3.14 to -0.06)	0.042	-0.76 (-2.30 to 0.79)	0.336	-0.84 (-2.42 to 0.73)	0.294
28 weeks	-3.37 (-4.96 to -1.77)	<0.001	-2.42 (-4.00 to -0.83)	0.003	-0.95 (-2.64 to 0.74)	0.271
56 weeks	-3.07 (-4.66 to -1.48)	<0.001	-2.33 (-3.86 to -0.80)	0.003	-0.74 (-2.31 to 0.84)	0.358
**100% MICE**						
10 weeks	-1.71 (-3.25 to -0.17)	0.030	-0.76 (-2.33 to 0.82)	0.347	-0.95 (-2.60 to 0.69)	0.255
28 weeks	-3.58 (-5.23 to -1.93)	<0.001	-2.48 (-4.14 to -0.83)	0.003	-1.10 (-2.77 to 0.58)	0.198
56 weeks	-3.44 (-5.03 to -1.85)	<0.001	-2.38 (-3.94 to -0.82)	0.003	-1.06 (-2.62 to 0.49)	0.181

Table 5 Multiple Imputation: Fatigue Severity Scale

	**PEP vs UC**		**CBA vs UC**		**PEP vs CBA**	
	**Estimate**	**p value**	**Estimate**	**p value**	**Estimate**	**p value**

**Active:All base; TAU:All MICE**						
10 weeks	-0.19 (-0.46 to 0.08)	0.109	-0.04 (-0.30 to 0.23)	0.757	-0.15 (-0.41 to 0.10)	0.176
28 weeks	-0.35 (-0.62 to -0.08)	0.004	-0.14 (-0.41 to 0.13)	0.248	-0.21 (-0.47 to 0.04)	0.061
56 weeks	-0.43 (-0.69 to -0.17)	<0.001	-0.43 (-0.69 to -0.17)	<0.001	0.00 (-0.25 to 0.26)	0.981
**All base**						
10 weeks	-0.21 (-0.46 to 0.04)	0.060	-0.06 (-0.30 to 0.19)	0.620	-0.15 (-0.40 to 0.10)	0.168
28 weeks	-0.38 (-0.63 to -0.13)	0.001	-0.17 (-0.42 to 0.08)	0.130	-0.21 (-0.46 to 0.04)	0.056
56 weeks	-0.46 (-0.71 to -0.21)	<0.001	-0.46 (-0.71 to -0.21)	<0.001	0.00 (-0.25 to 0.25)	0.978
**50% base:50% MICE**						
10 weeks	-0.24 (-0.51 to 0.03)	0.045	-0.08 (-0.35 to 0.19)	0.497	-0.16 (-0.43 to 0.11)	0.191
28 weeks	-0.48 (-0.76 to -0.20)	<0.001	-0.23 (-0.50 to 0.05)	0.063	-0.26 (-0.54 to 0.03)	0.042
56 weeks	-0.58 (-0.86 to -0.30)	<0.001	-0.51 (-0.79 to -0.24)	<0.001	-0.07 (-0.35 to 0.21)	0.590
**33.3% base:66.7% MICE**						
10 weeks	-0.27 (-0.54 to 0.01)	0.029	-0.10 (-0.38 to 0.19)	0.449	-0.17 (-0.46 to 0.11)	0.173
28 weeks	-0.51 (-0.79 to -0.22)	<0.001	-0.24 (-0.52 to 0.05)	0.060	-0.27 (-0.56 to 0.01)	0.033
56 weeks	-0.61 (-0.90 to -0.33)	<0.001	-0.53 (-0.80 to -0.25)	<0.001	-0.09 (-0.37 to 0.19)	0.484
**25% base:75% MICE**						
10 weeks	-0.27 (-0.54 to 0.01)	0.029	-0.10 (-0.38 to 0.19)	0.436	-0.17 (-0.46 to 0.12)	0.189
28 weeks	-0.52 (-0.82 to -0.23)	<0.001	-0.25 (-0.54 to 0.03)	0.048	-0.27 (-0.57 to 0.03)	0.041
56 weeks	-0.64 (-0.93 to -0.35)	<0.001	-0.54 (-0.82 to -0.26)	<0.001	-0.10 (-0.39 to 0.19)	0.426
**100% MICE**						
10 weeks	-0.29 (-0.57 to 0.00)	0.025	-0.11 (-0.41 to 0.19)	0.413	-0.18 (-0.48 to 0.12)	0.182
28 weeks	-0.58 (-0.89 to -0.28)	<0.001	-0.28 (-0.58 to 0.01)	0.032	-0.30 (-0.60 to 0.01)	0.030
56 weeks	-0.71 (-0.99 to -0.42)	<0.001	-0.56 (-0.85 to -0.27)	<0.001	-0.14 (-0.43 to 0.14)	0.261

For Tables 4 and 5: Imputation using Multivariate imputation by chained equations (MICE) filled in missing values in multiple variables iteratively. For these models the base variables were included to inform the imputations were available as were dependent variables for both CFS and FSS at all timepoints and baseline variables age, gender, centre, baseline HADS depression subscale >10, marital status, employment, education, ethnicity, timepoint and whether that individual was affected at all by covid. To accommodate the level of missingness adequately 50 separate databases were generated. This process was conducted on each treatment arm separately. All these databases were incorporated into the models to assess the expected variation. In this case the models were multilevel glms adjusting for treatment by time interactions and HADS depression subscale >10 as fixed effects. Clusters denoted by the centres were adjusted as random effects along with the participants nested within each centre. The heterogeneity between treatment arms using therapists for clusters resulted in non-convergence of the models – hence the use of centres instead for the cluster effect.

Table 6 Comparison PEP vs CBA for primary and secondary outcomes

Outcome	PEP	CBA	PEP vs CBA	p value
**PRIMARY**				
**Chalder fatigue scale**				
Baseline	21·4 (5·6); 122	20·4 (5·8); 120		
10 weeks	16·5 (7·5); 91	17·2 (6·4); 95	-1.02 (-3.20 to 1.17)	0.296
28 weeks	14·9 (8·2); 79	15·7 (6·7); 88	-1.16 (-3.44 to 1.12)	0.256
56 weeks	16·5 (7·3); 88	16·7 (6·0); 103	-0.67 (-2.84 to 1.50)	0.489
**Fatigue Severity Scale**				
Baseline	5·5 (1·1); 121	5·4 (1·0); 117		
10 weeks	5·0 (1·2); 91	5·1 (1·1); 93	-0.16 (-0.49 to 0.17)	0.288
28 weeks	4·7 (1·4); 78	5·0 (1·1); 88	-0.31 (-0.65 to 0.04)	0.047
56 weeks	4·7 (1·5); 85	4·8 (1·3); 100	-0.06 (-0.39 to 0.27)	0.680
**Outcome**	**PEP**	**CBA**	**PEP vs CBA**	**p value**
**SECONDARY**				
**HADS Anxiety**				
Baseline	8·9 (4·4); 123	8·7 (4·5); 121		
10 weeks	8·6 (4·4); 89	8·6 (4·7); 92	-0.19 (-1.10 to 0.73)	0.691
28 weeks	7·5 (5·0); 77	7·9 (4·6); 88	-0.40 (-1.34 to 0.55)	0.409
56 weeks (P)	7·6 (4·9); 73	7·8 (4·4); 86	-0.40 (-1.35 to 0.56)	0.416
**HADS Depression**				
Baseline	6·7 (3·3); 123	6·5 (3·4); 121		
10 weeks	6·6 (3·7); 91	6·3 (3·7); 93	0.18 (-0.62 to 0.98)	0.662
28 weeks	5·4 (3·7); 78	5·9 (3·3); 88	-0.43 (-1.26 to 0.41)	0.314
56 weeks(P)	5·4 (3·6); 75	6·1 (3·4); 88	-0.56 (-1.40 to 0.28)	0.191
**SF-12 PCS**				

Baseline	34·7 (9·8); 117	34·1 (10·3); 116		
10 weeks	36·8 (9·7); 88	35·0 (10·0); 92	0.27 (-1.79 to 2.33)	0.798
28 weeks	36·3 (10·6); 73	34·6 (9·8); 85	-0.03 (-2.20 to 2.13)	0.975
56 weeks(P)	36·5 (10·6); 73	34·8 (10·6); 87	1.26 (-0.89 to 3.42)	0.251
**SF-12 MCS**				
Baseline	40·8 (11·3); 117	41·6 (11·2); 116		
10 weeks	42·3 (11·1); 88	44·3 (11·0); 92	-1.32 (-3.73 to 1.10)	0.286
28 weeks	45·3 (12·3); 73	45·0 (11·2); 85	1.42 (-1.12 to 3.96)	0.273
56 weeks(P)	44·8 (10·5); 73	45·3 (10·7); 87	0.33 (-2.20 to 2.86)	0.799
**Pain (NRS)**				
Baseline	5·9 (2·5); 121	5·7 (2·3); 119		
10 weeks	5·1 (2·7); 91	5·4 (2·4); 93	-0.29 (-0.97 to 0.39)	0.401
28 weeks	4·8 (2·9); 77	5·3 (2·2); 87	-0.49 (-1.19 to 0.21)	0.168
56 weeks(P)	5·2 (2·7); 79	5·3 (2·4); 93	-0.41 (-1.10 to 0.28)	0.248
**Sleep**				
Baseline	13·0 (5·3); 120	13·4 (4·9); 115		
10 weeks	12·1 (5·2); 89	11·8 (5·3); 91	-0.08 (-1.43 to 1.27)	0.907
28 weeks	10·6 (5·6); 78	11·0 (5·3); 87	-0.77 (-2.15 to 0.61)	0.275
56 weeks(P)	11·6 (5·9); 75	10·8 (5·8); 89	0.35 (-1.04 to 1.73)	0.625
**WPAI (overall work impairment)**				
Baseline	46·7 (26·8); 47	47·6 (26·0); 46		
10 weeks	44·0 (25·4); 37	46·3 (27·4); 30	-6.60 (-17.23 to 4.03)	0.224
28 weeks	38·0 (31·1); 33	46·5 (29·3); 29	-10.18 (-21.08 to 0.72)	0.067
56 weeks(P)	31·0 (21·6); 21	42·7 (23·9); 29	-11.56 (-23.56 to 0.43)	0.059
**Value Life Activities**				
Baseline	1·5 (0·8); 122	1·5 (0·8); 120		
10 weeks	1·3 (0·8); 90	1·4 (0·9); 93	-0.09 (-0.25 to 0.06)	0.234
28 weeks	1·2 (0·8); 78	1·4 (0·9); 88	-0.20 (-0.36 to -0.04)	0.014
56 weeks(P)	1·3 (0·9); 76	1·3 (0·9); 88	-0.10 (-0.26 to 0.06)	0.205

Data are shown as adjusted means (sd); n. Results are expressed as mean difference (md), 97.5% confidence intervals (97.5% CI) for the primary outcomes and 95% confidence intervals (95% CI) for the secondary outcomes. All were mixed effects models adjusted for treatment by time interactions, baseline outcome measure and baseline HADS depression subscale >10 both as fixed effects. The models also included random effects for any Therapist clustering allowing for any uneven therapist involvement between arms as well as for individuals nested within Therapists for the time interactions. However, note the Therapists were observed to have a major clustering effect only for the CBA arm and so 'Therapist' for the PEP and TAU arms were coded as being just the Centre each participant was from. Degrees of freedom were adjusted for the small number of clusters using the Kenward Rogers method. All missing baseline covariates were replaced by their Centre means as per the SAP.

PEP, personalised exercise programme; CBA, cognitive-behavioural approach; UC, usual

Table 7 Global Health status outcome and comparison of interventions

**Global Health Change from baseline**									
**Time/category**	**PEP**	**CBA**	**UC**	**PEP vs UC**	**p value**	**CBA vs UC**	**p value**	**PEP vs CBA**	**p value**
10 weeks	N= 95	N= 96	N= 102						
Very much better	1 (1.1)	-	3 (2.9)	0.44 (0.92 to 0.22)	0.028	0.50 (1.03 to 0.24)	0.062	0.88 (1.82 to 0.43)	0.739
Much better	7 (7.4)	7 (7.3)	8 (7.8)						
A little better	32 (33.7)	32 (33.3)	9 (8.8)						
No change	37 (38.9)	35 (36.5)	47 (46.1)						
A little worse	10 (10.5)	16 (16.7)	24 (23.5)						
Much worse	3 (3.2)	1 (1.0)	3 (2.9)						
Very much worse	-	1 (1.0)	1 (1.0)						
missing	5 (5.3)	4 (4.2)	7 (6.9)						
28 weeks	N= 86	N= 95	N= 93						
Very much better	6 (7.0)	2 (2.1)	-	0.17 (0.37 to 0.08)	<0.001	0.26 (0.56 to 0.12)	<0.001	0.64 (1.38 to 0.30)	0.259
Much better	12 (14.0)	13 (13.7)	4 (4.3)						
A little better	24 (27.9)	28 (29.5)	10 (10.8)						
No change	20 (23.3)	18 (18.9)	39 (41.9)						
A little worse	12 (14.0)	23 (24.2)	23 (24.7)						
Much worse	3 (3.5)	2 (2.1)	7 (7.5)						
Very much worse	1 (1.2)	1 (1.1)	-						
missing	8 (9.3)	8 (8.4)	10 (10.8)						
56 weeks (P)	N= 90	N= 103	N= 102						
Very much better	7 (7.8)	2 (1.9)	-	0.08 (0.17 to 0.04)	<0.001	0.26 (0.55 to 0.12)	<0.001	0.30 (0.65 to 0.14)	0.002
Much better	15 (16.7)	16 (15.5)	4 (3.9)						
A little better	21 (23.3)	22 (21.4)	8 (7.8)						
No change	18 (20.0)	15 (14.6)	31 (30.4)						
A little worse	16 (17.8)	24 (23.3)	35 (34.3)						
Much worse	4 (4.4)	14 (13.6)	13 (12.7)						
Very much worse	-	1 (1.0)	3 (2.9)						
missing	9 (10.0)	9 (8.7)	8 (7.8)						

Data are shown as n/N (%). Results are expressed as incidence rate ratios (IRR) with 95% confidence intervals (95% CI) and p-value. The model was a multilevel ordinal regression generalised linear model (glm), adjusted for treatment by time interactions and HADS depression subscale >10 at baseline as fixed effects. For this outcome, clustering was adjusted for by Centres with individuals nested within Centre's as random effects (clustering on therapists was not easily implemented for this outcome data type). Any missing baseline covariates were replaced by their Centre means as per the SAP.

PEP, personalised exercise programme; CBA, cognitive-behavioural approach; UC, usual care

Table 8 BRAF-MDQ individual domains and comparison of interventions

Outcome	PEP	CBA	UC	PEP vs UC	p value	CBA vs UC	p value	PEP vs CBA	p value
**BRAF-MDQ total score**									
Baseline	41·3 (14·2); 122	38·9 (13·2); 119	40·0 (12·2); 120						
10 weeks	34·4 (16·6); 91	34·8 (13·8); 94	35·4 (14·2); 95	-2.14 (-5.64 to 1.36)	0.231	0.39 (-3.16 to 3.94)	0.830	-2.53 (-6.58 to 1.52)	0.136
28 weeks	31·1 (17·4); 76	33·4 (14·2); 89	34·5 (13·8); 81	-5.07 (-8.76 to -1.38)	0.007	-0.76 (-4.42 to 2.89)	0.683	-4.31 (-8.49 to -0.13)	0.016
56 weeks	31·2 (18·4); 78	30·8 (14·9); 92	36·9 (14·2); 87	-6.99 (-10.63 to -3.34)	<0.001	-4.93 (-8.53 to -1.33)	0.007	-2.06 (-6.21 to 2.10)	0.262
**Physical domain**									
Baseline	16·1 (3·5); 122	16·1 (3·3); 120	16·6 (2·7); 120						
10 weeks	14·1 (4·7); 91	14·6 (4·3); 94	14·9 (4·4); 95	-0.51 (-1.79 to 0.78)	0.441	0.02 (-1.29 to 1.33)	0.973	-0.53 (-1.99 to 0.93)	0.434
28 weeks	12·7 (5·7); 78	13·8 (4·4); 89	15·3 (4·0); 83	-2.20 (-3.55 to -0.86)	0.001	-1.38 (-2.73 to -0.04)	0.044	-0.82 (-2.33 to 0.68)	0.238
56 weeks	13·2 (5·4); 78	13·2 (4·9); 92	15·6 (4·2); 87	-2.10 (-3.44 to -0.76)	0.002	-2.15 (-3.48 to -0.82)	0.001	0.05 (-1.45 to 1.55)	0.854
**Living domain**									
Baseline	10·6 (5·4); 121	9·7 (5·2); 120	10·4 (5·1); 120						
10 weeks	8·0 (5·8); 91	8·5 (5·1); 94	8·6 (5·3); 95	-0.62 (-1.89 to 0.65)	0.339	0.40 (-0.91 to 1.70)	0.553	-1.02 (-2.48 to 0.45)	0.130
28 weeks	7·2 (5·6); 78	8·0 (5·3); 88	8·2 (5·3); 81	-1.08 (-2.42 to 0.26)	0.113	0.24 (-1.11 to 1.59)	0.727	-1.32 (-2.83 to 0.19)	0.059
56 weeks	7·3 (6·2); 76	7·3 (5·4); 89	9·3 (5·8); 85	-1.97 (-3.30 to -0.63)	0.004	-0.89 (-2.23 to 0.44)	0.190	-1.07 (-2.59 to 0.44)	0.134
**Cognition domain**									
Baseline	8·1 (4·1); 121	7·3 (4·0); 119	7·0 (3·6); 120						
10 weeks	7·0 (4·5); 90	6·4 (3·7); 93	6·3 (3·9); 95	-0.17 (-1.06 to 0.71)	0.703	-0.18 (-1.02 to 0.67)	0.680	0.00 (-0.91 to 0.92)	0.996
28 weeks	6·0 (4·4); 78	6·5 (4·0); 88	6·1 (3·8); 82	-0.95 (-1.88 to -0.02)	0.046	0.16 (-0.72 to 1.03)	0.724	-1.11 (-2.06 to -0.15)	0.014
56 weeks	6·2 (4·6); 77	5·9 (3·9); 91	6·6 (3·9); 86	-1.18 (-2.10 to -0.25)	0.013	-0.85 (-1.71 to 0.01)	0.054	-0.33 (-1.28 to 0.62)	0.447
**Emotion domain**									
Baseline	6·5 (3·4); 121	5·8 (3·2); 120	6·0 (3·3); 120						
10 weeks	5·3 (3·7); 90	5·4 (3·3); 94	5·5 (3·4); 95	-0.59 (-1.36 to 0.19)	0.137	-0.07 (-0.85 to 0.72)	0.864	-0.52 (-1.37 to 0.34)	0.192
28 weeks	4·6 (3·7); 78	5·0 (3·4); 88	5·4 (3·5); 82	-1.23 (-2.05 to -0.42)	0.003	-0.38 (-1.20 to 0.43)	0.354	-0.85 (-1.73 to 0.03)	0.040
56 weeks	4·4 (4·1); 77	4·4 (3·4); 91	5·6 (3·6); 86	-1.72 (-2.53 to -0.91)	<0.001	-1.03 (-1.84 to -0.23)	0.011	-0.69 (-1.57 to 0.19)	0.096

Data are shown as adjusted means (sd); n. Results are expressed as mean difference (md), 95% confidence intervals (95% CI) since this is a secondary outcome, and p values. All were mixed effects models adjusted for treatment by time interactions, baseline outcome measure and baseline HADS depression subscale >10 both as fixed effects. The models also included random effects for any Therapist clustering allowing for any uneven therapist involvement between arms as well as for individuals nested within Therapists for the time interactions. However, note the Therapists were observed to have a major clustering effect only for the CBA arm and so 'Therapist' for the PEP and TAU arms were coded as being just the Centre each participant was from. Degrees of freedom were adjusted for the small number of clusters using the Kenward Rogers method. All missing baseline covariates were replaced by their Centre means as per the SAP.

PEP, personalised exercise programme; CBA, cognitive-behavioural approach; UC, usual care

Additional post-hoc analyses

Effect of compliance

Compliance is defined by a participant attending 3 or more sessions. Data are shown as adjusted means (sd); n.
Both outcome compliance models are conducted as two stage standard least square regressions adjusted. In this case if compliance has been satisfied or not is treated as an endogenous variable as part of the first stage. This then is incorporated into the second stage that assesses the treatment effect on outcomes of interest. This second stage as with the main analyses adjusts for the baseline outcome measure and each participants' baseline HADS depression subscale >10 both as fixed effects. The models also included random effects for any Centre clustering. Any missing baseline covariates were replaced by their Centre means as per the SAP. Clustering effects were adjusted for at Centre level using a robust variance structure (Heterogeneous therapists between arms was not easily accommodated in these models)

Table 9 Primary Outcomes summaries and CACE Models estimates to incorporating the effect of compliance

Outcome	PEP	CBA	UC	PEP vs UC (97.5% CI)	p value	CBA vs UC (97.5% CI)	p value
**Chalder fatigue scale**							
Baseline	21·4 (5·6); 122	20·4 (5·8); 120	20·7 (5·2); 120				
10 weeks	16·5 (7·5); 91	17·2 (6·4); 95	17·9 (6·2); 94				
28 weeks	14·9 (8·2); 79	15·7 (6·7); 88	18·4 (5·7); 82				
56 weeks	16·5 (7·3); 88	16·7 (6·0); 103	19·2 (5·9); 100	-4.44 (-5.66, -3.21)	<0.001	-2.64 (-4.39, -0.88)	0.001
**Fatigue Severity Scale**							
Baseline	5·5 (1·1); 121	5·4 (1·0); 117	5·5 (0·9); 119				
10 weeks	5·0 (1·2); 91	5·1 (1·1); 93	5·3 (1·1); 95				
28 weeks	4·7 (1·4); 78	5·0 (1·1); 88	5·3 (1·1); 83				
56 weeks	4·7 (1·5); 85	4·8 (1·3); 100	5·4 (1·1); 99	-0.71 (-1.06, -0.36)	<0.001	-0.54 (-0.85, -0.22)	<0.001

Sub-group analyses

The Following subgroup tables are each modelled using treatment x (appropriate) subgroup interactions. In addition, all models were adjusted for their baseline outcome measure and their baseline HADS depression subscale >10 both as fixed effects. The models also included random effects for any Therapist clustering allowing for any uneven therapist involvement between arms as well as for individuals nested within Therapists. However, note the Therapists were observed to have a major clustering effect only for the CBA arm and so 'Therapist' for the PEP and TAU arms were coded as being just the Centre's participating from.
Degrees of freedom were adjusted for the small number of clusters using the Kenward Rogers method. For ease of interpretation while for each subgroup the covariates are all included in the model, the treatment effects here are split for each subgroup category along with their respective summaries at each time point shown as adjusted means (sd); n.

Disease Type Impact

Table 10 Post hoc Sub-Group analyses for Disease Type impact: Chalder Fatigue Scale

Sub-group	PEP	CBA	UC	PEP vs UC	p value	CBA vs UC	p value
**RA**							
Baseline	21.3(5.0): 67	20.2(5.6): 67	19.6(4.8): 68				
10 weeks	16.8 (6.1):49	16.5 (5.8):54	18.4 (5.9):55	-2.28 (-5.00 to 0.45)	0.061	-2.15 (-4.74 to 0.44)	0.062
28 weeks	15.4 (7.5):44	14.8 (6.3):48	18.1 (5.4):49	-3.12 (-5.98 to -0.27)	0.014	-3.15 (-5.86 to -0.45)	0.009
56 weeks (P)	16.6 (6.6):52	16.5 (5.8):59	19.0 (6.1):58	-3.04 (-5.70 to -0.39)	0.010	-2.64 (-5.16 to -0.12)	0.019
**SpA**							
Baseline	19.9(6.4): 25	21.4(6.8): 24	20.1(4.5): 21				
10 weeks	15.2 (7.7):20	19.9 (8.1):16	15.5 (4.2):16	-0.66 (-5.20 to 3.87)	0.743	3.09 (-1.49 to 7.67)	0.130
28 weeks	12.4 (6.9):13	17.6 (8.8):14	16.3 (3.8):14	-4.00 (-9.20 to 1.19)	0.084	-0.91 (-5.75 to 3.93)	0.672
56 weeks (P)	14.2 (7.7):17	15.6 (7.2):18	19.1 (5.5):19	-4.98 (-9.56 to -0.39)	0.015	-4.83 (-9.14 to -0.52)	0.012
**CTD**							
Baseline	22.7(5.9): 26	20.4(5.5): 26	23.6(5.9): 25				
10 weeks	17.2 (9.7):18	17.3 (6.3):22	17.7 (7.5):19	-0.22 (-4.72 to 4.29)	0.914	0.84 (-3.28 to 4.95)	0.649
28 weeks	15.9 (10.1):18	16.0 (6.3):23	20.7 (7.6):15	-4.87 (-9.56 to -0.17)	0.020	-3.91 (-8.20 to 0.38)	0.041
56 weeks (P)	17.4 (7.7):16	18.2 (6.1):23	19.7 (6.4):19	-1.67 (-6.33 to 2.98)	0.421	-0.19 (-4.28 to 3.90)	0.916
**Other**							
Baseline	23.8(5.9): 4	16.3(1.5): 3	23.2(4.9): 6				
10 weeks	17.5 (12.6):4	14.7 (4.5):3	21.3 (8.7):4	-3.68 (-13.40 to 6.05)	0.397	-1.70 (-11.74 to 8.34)	0.704

Sub-group	PEP	CBA	UC	PEP vs UC	p value	CBA vs UC	p value
28 weeks	12.5 (10.8):4	18.4 (3.9):3	21.5 (4.4):4	-8.93 (-18.65 to 0.80)	0.040	1.82 (-8.23 to 11.86)	0.685
56 weeks (P)	21.7 (13.6):3	16.7 (2.1):3	19.8 (5.1):4	0.50 (-10.03 to 11.02)	0.916	1.80 (-8.24 to 11.84)	0.688

Table 11 Post hoc Sub-Group analyses for Disease type: Fatigue Severity Scale

Sub-group	PEP	CBA	UC	PEP vs UC	p value	CBA vs UC	p value
**RA**							
Baseline	5.4(1.1): 66	5.3(1.1): 64	5.4(0.9): 67				
10 weeks	5.0 (1.2):49	5.0 (1.1):52	5.4 (1.0):56	-0.32 (-0.73 to 0.09)	0.084	-0.30 (-0.70 to 0.10)	0.096
28 weeks	4.6 (1.3):43	4.8 (1.1):48	5.3 (1.0):48	-0.52 (-0.95 to -0.09)	0.007	-0.36 (-0.78 to 0.06)	0.053
56 weeks (P)	4.8 (1.5):51	4.7 (1.3):58	5.4 (1.1):58	-0.61 (-1.02 to -0.21)	0.001	-0.61 (-1.00 to -0.22)	<0.001
**SpA**							
Baseline	5.2(1.3): 25	5.6(1.0): 24	5.6(0.9): 21				
10 weeks	4.8 (1.2):20	5.7 (1.0):16	4.9 (1.2):16	0.14 (-0.55 to 0.84)	0.641	0.67 (-0.05 to 1.40)	0.038
28 weeks	4.1 (1.7):13	5.4 (1.0):14	5.2 (0.8):15	-0.72 (-1.50 to 0.06)	0.038	-0.04 (-0.80 to 0.71)	0.899
56 weeks (P)	4.3 (1.5):16	4.8 (1.2):16	5.3 (1.1):18	-0.74 (-1.45 to -0.02)	0.021	-0.45 (-1.16 to 0.26)	0.157
**CTD**							
Baseline	5.8(1.1): 26	5.8(0.7): 26	5.6(1.0): 25				
10 weeks	5.3 (1.4):18	5.2 (0.8):22	5.3 (1.2):19	-0.27 (-0.95 to 0.42)	0.388	-0.14 (-0.79 to 0.50)	0.624
28 weeks	5.3 (1.2):18	5.3 (1.1):23	5.5 (1.6):16	-0.42 (-1.13 to 0.29)	0.183	-0.26 (-0.92 to 0.40)	0.375
56 weeks (P)	5.1 (1.3):16	5.1 (1.3):23	5.5 (1.2):19	-0.55 (-1.26 to 0.16)	0.083	-0.47 (-1.11 to 0.17)	0.100
**Other**							
Baseline	5.6(1.2): 4	4.7(0.2): 3	5.9(1.0): 6				
10 weeks	4.9 (1.8):4	4.7 (0.7):3	6.1 (0.6):4	-1.06 (-2.56 to 0.44)	0.113	-0.38 (-2.01 to 1.25)	0.603
28 weeks	4.4 (2.0):4	4.9 (1.4):3	5.4 (1.1):4	-0.89 (-2.39 to 0.60)	0.181	0.50 (-1.13 to 2.13)	0.490
56 weeks (P)	4.6 (3.0):2	3.0 (0.7):3	5.8 (0.4):4	-0.99 (-2.80 to 0.82)	0.219	-1.73 (-3.36 to -0.10)	0.017

Covid impact

Table 12 Post hoc Sub-Group analyses for COVID impact: Chalder Fatigue Scale

Sub-group	PEP	CBA	UC	PEP vs UC	p value	CBA vs UC	p value
CD							
Baseline	21.5(5.5): 44	21.0(5.8): 42	21.0(5.4): 38				
10 weeks	16.4 (6.8):31	18.7 (6.4):33	17.8 (6.1):31	-2.19 (-5.64 to 1.27)	0.156	0.92 (-2.35 to 4.19)	0.529
28 weeks	17.3 (9.1):30	16.4 (6.8):32	18.4 (6.2):28	-1.92 (-5.47 to 1.63)	0.225	-1.94 (-5.30 to 1.42)	0.195
56 weeks (P)	16.6 (7.1):28	17.1 (6.6):39	18.7 (5.7):33	-2.68 (-6.19 to 0.84)	0.088	-1.41 (-4.53 to 1.70)	0.309
non-CD							
Baseline	21.4(5.7): 78	20.1(5.8): 78	20.5(5.2): 82				
10 weeks	16.6 (7.9):60	16.4 (6.3):62	17.9 (6.2):63	-1.48 (-3.98 to 1.02)	0.184	-1.53 (-3.94 to 0.88)	0.154
28 weeks	13.4 (7.2):49	15.3 (6.6):56	18.5 (5.5):54	-5.11 (-7.80 to -2.41)	<0.001	-3.13 (-5.66 to -0.61)	0.005
56 weeks (P)	16.4 (7.5):60	16.5 (5.7):64	19.4 (6.1):67	-3.26 (-5.73 to -0.79)	0.003	-2.84 (-5.20 to -0.47)	0.007

Table 13 Post hoc Sub-Group analyses COVID impact: Fatigue Severity Scale

Sub-group	PEP	CBA	UC	PEP vs UC	p value	CBA vs UC	p value
CD							
Baseline	5.5(1.1): 44	5.7(0.9): 42	5.4(0.8): 38				
10 weeks	4.9 (1.4):31	5.4 (0.7):33	5.2 (1.1):31	-0.41 (-0.93 to 0.12)	0.086	0.01 (-0.51 to 0.53)	0.972
28 weeks	4.9 (1.5):30	5.3 (0.9):32	5.1 (1.2):29	-0.34 (-0.88 to 0.20)	0.156	-0.01 (-0.54 to 0.52)	0.959
56 weeks (P)	4.5 (1.7):25	4.8 (1.3):36	5.3 (1.2):31	-0.98 (-1.53 to -0.42)	<0.001	-0.70 (-1.21 to -0.19)	0.002
non-CD							
Baseline	5.4(1.2): 77	5.3(1.1): 75	5.6(1.0): 81				
10 weeks	5.1 (1.1):60	5.0 (1.2):60	5.4 (1.1):64	-0.19 (-0.56 to 0.19)	0.266	-0.16 (-0.54 to 0.22)	0.334
28 weeks	4.5 (1.4):48	4.9 (1.2):56	5.4 (1.1):54	-0.67 (-1.08 to -0.27)	<0.001	-0.36 (-0.75 to 0.03)	0.041
56 weeks (P)	4.8 (1.4):60	4.8 (1.3):64	5.5 (1.1):68	-0.49 (-0.87 to -0.12)	0.003	-0.51 (-0.88 to -0.14)	0.002

Gender impact

Table 14 Post hoc Sub-Group analyses for Gender impact: Chalder Fatigue Scale

Sub-group	PEP	CBA	UC	PEP vs UC	p value	CBA vs UC	p value
Female							
Baseline	22.2(5.3): 96	20.3(5.9): 83	21.1(5.5): 92				
10 weeks	16.8 (7.6):71	17.6 (6.1):69	18.4 (5.9):72	-2.30 (-4.59 to -0.01)	0.025	-0.52 (-2.78 to 1.74)	0.607
28 weeks	15.3 (8.8):65	15.5 (6.3):67	19.1 (5.5):63	-4.43 (-6.83 to -2.04)	<0.001	-3.39 (-5.72 to -1.06)	0.001
56 weeks (P)	17.0 (7.6):68	16.9 (5.7):75	19.7 (6.1):76	-3.28 (-5.57 to -0.99)	0.001	-2.38 (-4.58 to -0.18)	0.015
Male							
Baseline	18.3(5.4): 26	20.6(5.7): 37	19.4(4.2): 28				
10 weeks	15.6 (7.0):20	16.2 (7.0):26	16.2 (6.9):22	0.32 (-3.90 to 4.54)	0.864	-0.81 (-4.55 to 2.94)	0.630
28 weeks	12.8 (3.5):14	16.4 (8.0):21	16.2 (6.0):19	-2.10 (-6.88 to 2.68)	0.325	-0.41 (-4.45 to 3.63)	0.819
56 weeks (P)	14.7 (6.0):20	16.3 (7.0):28	17.7 (5.4):24	-2.25 (-6.41 to 1.90)	0.224	-2.07 (-5.69 to 1.55)	0.201

Table 15 Post hoc Sub-Group analyses for Gender impact: Fatigue Severity Scale

Sub-group	PEP	CBA	UC	PEP vs UC	p value	CBA vs UC	p value
Female							
Baseline	5.7(1.0): 95	5.5(1.0): 81	5.5(0.9): 91				
10 weeks	5.2 (1.2):71	5.2 (1.1):67	5.4 (1.0):73	-0.30 (-0.65 to 0.05)	0.055	-0.21 (-0.57 to 0.14)	0.178
28 weeks	4.8 (1.4):64	5.1 (1.0):67	5.4 (1.0):64	-0.56 (-0.92 to -0.20)	0.001	-0.24 (-0.60 to 0.12)	0.137
56 weeks (P)	4.9 (1.4):66	4.8 (1.2):73	5.5 (1.0):75	-0.73 (-1.09 to -0.38)	<0.001	-0.74 (-1.09 to -0.40)	<0.001
Male							
Baseline	4.7(1.3): 26	5.3(1.1): 36	5.4(0.9): 28				
10 weeks	4.4 (1.1):20	5.1 (1.0):26	5.0 (1.2):22	-0.17 (-0.82 to 0.48)	0.555	0.21 (-0.39 to 0.80)	0.436
28 weeks	4.0 (1.3):14	4.8 (1.4):21	5.2 (1.3):19	-0.49 (-1.22 to 0.23)	0.127	-0.26 (-0.90 to 0.38)	0.358
56 weeks (P)	4.3 (1.5):19	4.8 (1.6):27	5.1 (1.3):24	-0.33 (-0.98 to 0.31)	0.246	-0.09 (-0.67 to 0.49)	0.723

Table 16 Training and Supervision time

Time in hours (mean±SD)	PEP	CBA
Training	5.71±2.81	16.55±2.02
Supervision	8.00±3.65	8.00±0
Total	13.71±4.76	24.55±2.02

Table 17 Trial amendments

Amendment Number	Date Submitted	Purpose of Amendment	Version/Date of amended documents	
			New	Old
01	23 June 2017	- reflect introduction of changes in language to enhance clarity<br><br>- moderate changes to the protocol which were recommended after review by the Trial Steering Committee and patient support groups	Variable, see letter of favourable opinion for details	Variable, see letters of Favourable opinion with additional conditions 8.3.17 and Acknowledgement of additional conditions met 20.3.17
02	17 Aug 2017	- optional MRI brain scan which will be offered to recruited LIFT participants	Protocol v4 (31 Aug 2017)<br><br>PIS for MRI v2 (31 Aug 2017)	Protocol v2 (23 June 2017), v3 (25 July 2017; submitted<br><br>PIS for MRI v1 (25 July 2017; submitted)

Amendment Number	Date Submitted	Purpose of Amendment	Version/Date of amended documents	
			New	Old
			Consent for MRI v1 (25 July 2017)	
03	21 Nov 2017	- to open up recruitment to all inflammatory rheumatic diseases This change also ameliorates the need to collect of classification criteria and disease specific activity scores for all IRDs, except RA<br>- to make these measure non-mandatory apart from RA<br>- submission of new documents (database user guides for participants) to encourage use of the study database for direct data collection	Protocol v5 (21 Nov 2017)<br><br>PIS v4 (21 Nov 2017)<br><br>General notification letter PEP intervention v2 (09 Nov 2017)<br><br>Screening questions and consent v2 (17 Nov 2017)<br><br>LIFT study database Participant Login v1 (09 Nov 2017)<br><br>LIFT study database Participant Data Entry General v1 (09 Nov 2017)<br><br>LIFT study database Participant Cost Diaries v1 (09 Nov 2017)<br><br>LIFT study database PEP Participant Activity and Exercise Diary v1 (09 Nov 2017)<br><br>LIFT study database PEP Participant Downloads v1 (09 Nov 2017)<br><br>LIFT study database PEP Participant Goals v1 (09 Nov 2017)	Protocol v4 (31 Aug 2017)<br><br>PIS v3 (23 Jun 2017)<br><br>General notification letter PEP intervention v1 (18 May 2017)<br><br>Screening questions and consent v1 (17 Feb 2017)
04	21/02/2018	-changes to the protocol and supporting documents (screening questions, invitation cover letter, questionnaire visit 4 telephone script, study poster) to increase recruitment into the study and collection of primary outcomes at 56 weeks.<br>-Small changes to the wording of the screening questionnaire and consent form as well as the cover	Protocol v6 (08 Feb 2018)<br><br>Screening questions and consent v3 (09 Jan 2018)<br><br>Invitation Cover letter Standard v2 (01 Feb 2018)	Protocol v5 (21 Nov 2017)<br><br>Screening questions and consent v2 (17 Nov 2017)<br><br>Invitation Cover letter Standard v1 (17 Feb 2017)

Amendment Number	Date Submitted	Purpose of Amendment	Version/Date of amended documents	
			New	Old
		letter were made in response to feedback from the research nurses on the study sites to improve contact with potentially patients, reduce research nurse burden and thus facilitate recruitment.<br>-Rewording of the health care cost diary reminders and change of frequency was done in response to feedback given by current study participants.-	Invitation Cover letter TDF v2 (01 Feb 2018)<br><br>Courtesy Reminder health care cost diary v3 (01 Feb 2018)<br><br>Study poster v1 (21 Feb 2018)<br><br>Questionnaire visit 4 telephone script v1 (08 Feb 2018)	Invitation Cover letter TDF v1 (17 Feb 2017)<br><br>Courtesy Reminder health care cost diary v2 (02 June 2017)
05	23/02/2018	Addition of new study sites<br>Newcastle<br>Guy's and St Thomas	n/a	n/a
06	12/03/2018	Addition of new study sites<br>Stoke-on-Trent<br>Gateshead<br>Lincolnshire and Goole<br>Solent	n/a	n/a
07	19/03/2018	Use of DaSH safehaven in NHS Grampian to identify patients by local direct care team (DaSH275, CG-2018-18)	n/a	n/a
08	24/05/2018	Privacy notice due to GDPR using HRA approved text. Non-substantial and non-notifiable amendment	Privacy notices for all current sites for LIFT and LIFT MRI study	n/a
09	26/06/2018	New study site for MRI sub-study (NHS Greater Glasgow and Clyde)<br>Extension for 6 months until 31.07.2020 (grant) and recruitment until 31.03.2019	Study protocol v7 (25 Jun 2018)<br><br>Participant Information Sheet MRI sub-study v3 (25 Jun 2018)	Study protocol v6 (8 Feb 2018)<br><br>Participant Information Sheet MRI sub-study v2 (31 Aug 2017)
10	05/12/2018	Change of CI, Amendment to Qualitative Evaluation study	Study protocol v8 (23 Nov 2018)<br>Additional paperwork, see documentation of amendment	Study protocol v7 (25 Jun 2018)
11	28/02/2019	Extension for 6 months until 31.01.2021 (grant) and recruitment until 30.09.2019	n/a	
12	14/05/2019	Option to invite LIFT study therapists to take part in the Qualitative Evaluation sub-study whilst they may still see participants but have gained sufficient experience.	LIFT study protocol v9<br><br>LIFT qualitative study cover letter tutor CBA v3	LIFT study protocol v8<br><br>LIFT qualitative study cover letter tutor CBA v2

Amendment Number	Date Submitted	Purpose of Amendment	Version/Date of amended documents	
			New	Old
			LIFT qualitative study cover letter tutor PEP v3<br><br>LIFT qualitative study Therapist Information Sheet CBA v3<br><br>LIFT qualitative study Therapist Information Sheet PEP v3	LIFT qualitative study cover letter tutor PEP v2<br><br>LIFT qualitative study Therapist Information Sheet CBA v2<br><br>LIFT qualitative study Therapist Information Sheet PEP v2
13	16/03/2020	Option to perform remote LIFT assessment visits 3 and 4 by Trial Office should the local research nurse team not be able to do a visit in person or remote due to COVID-19	LIFT study protocol v10<br><br>Remote visit for data collection at Assessment visit 3 and 4 by Trial Office v1	LIFT study protocol v9
14	20/10/2020	Transfer of optional blood samples to Glasgow	LIFT study protocol v11	LIFT study protocol v10
